# Noncoding RNAs in apoptosis: identification and function

**DOI:** 10.3906/biy-2109-35

**Published:** 2021-11-14

**Authors:** Özge TÜNCEL, Merve KARA, Bilge YAYLAK, İpek ERDOĞAN, Bünyamin AKGÜL

**Affiliations:** Non-coding RNA Laboratory, Department of Molecular Biology and Genetics, Faculty of Science, İzmir Institute of Technology, İzmir, Turkey

**Keywords:** Apoptosis, noncoding RNA, microRNA, long noncoding RNA, circular RNA

## Abstract

Apoptosis is a vital cellular process that is critical for the maintenance of homeostasis in health and disease. The derailment of apoptotic mechanisms has severe consequences such as abnormal development, cancer, and neurodegenerative diseases. Thus, there exist complex regulatory mechanisms in eukaryotes to preserve the balance between cell growth and cell death. Initially, protein-coding genes were prioritized in the search for such regulatory macromolecules involved in the regulation of apoptosis. However, recent genome annotations and transcriptomics studies have uncovered a plethora of regulatory noncoding RNAs that have the ability to modulate not only apoptosis but also many other biochemical processes in eukaryotes. In this review article, we will cover a brief summary of apoptosis and detection methods followed by an extensive discussion on microRNAs, circular RNAs, and long noncoding RNAs in apoptosis.

## 1. Introduction

Historically, it was assumed by scientists that cell death is a natural consequence of life without any genetic basis. However, the accumulating evidence clearly indicates that cell death can be intervened both pharmacologically and genetically, paving the way for its use in health and disease (Fuchs and Steller, 2015). The Nomenclature Committee on Cell Death (NCCD) released a report in 2018, proposing two major cell death modes (1) accidental cell death (ACD) and (2) regulated cell death (RCD) (Galluzzi et al., 2018). ACD comes about physico-chemically or mechanically and, thus, has no application in clinics due to its irreversible nature. On the other hand, the evolutionarily conserved and reversible RCD has an essential function in several key cellular processes such as elimination of excess cells or life-threatening antigens (Hotchkiss et al., 2009; Chao et al., 2012; Ouyang et al., 2012; Singh et al., 2012). The rate of RCD is highly critical in maintaining organismal homeostasis. Any decline in the rate of cell death may result in cancer and autoimmune diseases while an elevation in its rate may lead to neurodegenerative diseases or AIDS. The cellular function of RCD is not confined to diseases as it orchestrates numerous physiological processes such as morphogenesis and organogenesis (Arya and White, 2015).

The first example of programmed cell death (PCD) was reported in 1965 in a study involving the development of muscle subsegments (Lockshin and Williams, 1965). The term apoptosis, which means “falling off” in Greek, was then coined in 1972 to describe the characteristics of a dying cell such as cell shrinkage, chromatin condensation, nuclear fragmentation, blebbing, and apoptotic bodies (Kerr et al., 1972). Since the first morphological description of cell death in 1965, it took nearly 27 years to characterize an apoptotic protein at the molecular level (Thornberry et al., 1992). Subsequent studies have led to the identification of three major types of cell death although it is sometimes difficult to draw the boundaries among them unequivocally: apoptosis, autophagy, and necrosis (Eisenberg-Lerner et al., 2009; Hotchkiss et al., 2009; Maghsoudi et al., 2012; Berghe et al., 2014; Teng and Hardwick, 2015). Currently twelve major cell death subroutines have been characterized in mammals based on the types of changes in the intracellular or extracellular microenvironment (Galluzzi et al., 2018).

In this review, we will succinctly describe major apoptotic pathways followed by the coverage of three major types of noncoding RNAs (ncRNAs) that modulate apoptosis, namely microRNAs (miRNAs), circular RNAs (circRNAs) and long noncoding RNAs (lncRNAs).

## 2. Apoptosis

Apoptosis is a type of programmed cell death performed by activation of a family of cysteine proteases known as caspases. Apoptosis takes place in an ordered chain reaction triggered by various intra- or extracellular stimuli such as ligation of cell surface receptors, treatment with cytotoxic drugs or irradiation (Blank and Shiloh, 2007). In response to an apoptotic stimulus, cells exhibit the hallmarks of apoptosis depicted as shrinkage in cellular volume and chromatin condensation, which is followed by membrane blebbing, nucleus fragmentation and, eventually, apoptotic body formation. These morphological features are cell type-, stimulus- and apoptotic stage-dependent. During apoptotic processes, cells, which are defined as apoptotic bodies henceforth, are rapidly removed from the site of action through ATP and UTP release, proceeding with phosphatidylserine presentation for phagocytosis by macrophages (Nirmala and Lopus, 2020). Maintenance of plasma membrane integrity prevents the release of the intracellular content as well as any potential inflammatory reactions (Elmore, 2007). In case of inefficient removal of the apoptotic cells, secondary necrosis may arise, leading to chronic inflammatory diseases such as type 2 diabetes, atherosclerosis, chronic obstructive pulmonary disease (COPD), cystic fibrosis, asthma as well as, Parkinson’s, Alzheimer’s, and Huntington’s disease (Szondy et al., 2014).

### 2.1. Pathways of apoptosis

**Figure 1:**
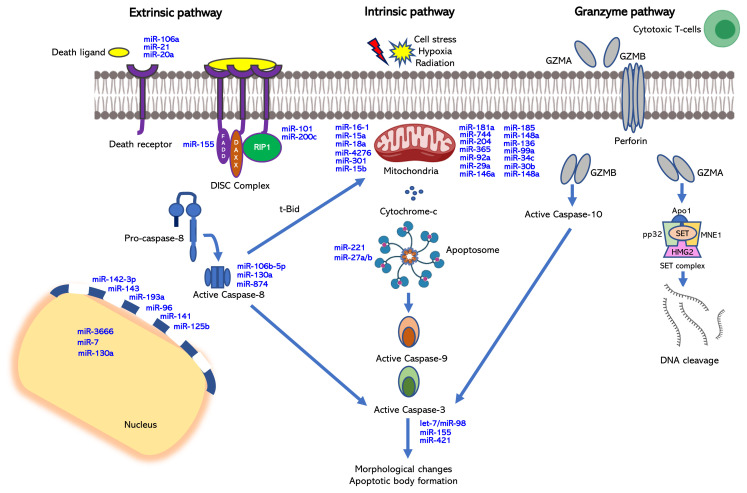
Three pathways of apoptosis. The extrinsic signalling pathway is initiated through the binding of a ligand to transmembrane receptors. The death domain plays a critical role in transmitting the death signal from the cell surface into the cell through caspase-8. External stimuli such as cell stress, hypoxia, and radiation trigger the intrinsic pathway that causes cytochrome-c release from mitochondria. Cytotoxic T-cells are the main controllers for the granzyme pathway, which results in caspase-10 activation. Granzyme B can activate caspases in the targeted cell.

As being committed in a fashion of a cascade of events, apoptosis proceeds through the phases of initiation, intracellular response, cell fragmentation, and phagocytosis (Afford and Randhawa, 2000). Based on initiator signals and executory processes, the pathways of apoptosis are classified into three subgroups: extrinsic, intrinsic, and perforin/granzyme pathways (Figure 1). First two pathways crosstalk with each other during caspase activation in the execution phase, and all three pathways share the same termination module in the end (Igney and Krammer, 2002).

A specific interaction between a ligand and transmembrane death receptors, such as tumour necrosis factor receptor 1 (TNFR1) and Fas receptor (FasR), which are members of the tumor necrosis factor (TNF) receptor gene superfamily, initiates apoptosis through the extrinsic signalling pathway. The ligand-receptor interaction activates the sequential events of recruitment of cytosolic adaptor proteins harboring death domains, death domain association with procaspase-8, formation of death-inducing signal complex (DISC) and activation of procaspase-8 to initiator caspase-8. Subsequently, executioner caspases (caspases-3, - 6 and - 7) are activated to complete the process (Blank and Shiloh, 2007; Elmore, 2007).

The intrinsic pathway is initiated through stress stimuli such as direct DNA damage, hypoxia, and survival factor deprivation, and proceeds with the loss of mitochondrial membrane potential, resulting in an alteration in the permeability of mitochondria. Cytochrome c is then released from the intermembrane space into the cytosol and interacts with proapoptotic cytosolic factor Apaf-1 to form the apoptosome, which activates caspase-9 and -3, consecutively (Bender and Martinou, 2013). Synchronously, inhibitor of apoptosis proteins (IAP) is inhibited by Smac/DIABLO and HtrA2/Omi to promote apoptosis. DNA fragmentation is a late event of apoptosis that is induced by apoptosis inducing factor (AIF) and endonuclease G (EndoG) released from the mitochondria (Nirmala and Lopus, 2020). Both extrinsic and intrinsic pathways are linked to each other via Bid, a pro-apoptotic member of the Bcl-2 family (Igney and Krammer, 2002). Translocation of Bid to the mitochondria and its cleavage into the truncated Bid (t-Bid) by caspase-8 permeabilizes the mitochondrial membrane, resulting in the amplification of apoptotic signals (Billen et al., 2008).

The perforin/granzyme pathway, which is specialized for infected cells, is executed by cytotoxic T-lymphocytes and natural killer cells (Igney and Krammer, 2002; Nirmala and Lopus, 2020). Immune cells in the infection site release perforin to disrupt the membrane integrity of the target cells. The resulting pores then induce apoptosis with the action of granzyme A and B, a group of serine proteases. Granzyme A causes DNA fragmentation in a caspase-independent manner by cleaving the SET complex, which inhibits NM23-H1, a tumor suppressor gene (Elmore, 2007). Granzyme B activates procaspase-10 and contributes to the intrinsic pathway via caspase-9 and -3.

### 2.2. Detection methods of apoptosis

Apoptotic features can be distinguished via various methods based on cell morphology, DNA structure, biochemical and electrochemical properties of apoptotic cells either real-time or at an end point (Table 1). Recent advances in the detection methods have facilitated data acquisition with less intervention to the population of interest, which mitigates the extent of a bias during data processing.

### 2.3. Electron microscopy

Morphological features of apoptosis can be monitored by electron microscopy, either by scanning (SEM) or transmission electron microscopy (TEM). SEM provides topographic data depicting cell surface alterations by using a focused beam of high energy electrons (Burattini and Falcieri, 2013). Nonhazardous electron bombardment on the surface allows to obtain micrographs from a broader perspective but in lower resolution when compared with TEM, which is considered as the gold standard (Elmore, 2007). Instead of utilizing reflected electrons, the image in TEM is obtained by the electron passage through the specimen, which provides a higher resolution of inner structures. On the other hand, the specimen preparation is more laborious in TEM, and the field of view is much smaller than SEM. Unfortunately, none of these methods can be applied to real-time imaging as they serve as endpoint assays (Martinez et al., 2010). Acquisition of precise data on the characteristic features of the early stages of apoptosis is difficult to attain due to the dynamic nature of apoptosis and stage-based variations among cell types (Otsuki et al., 2003).

### 2.4. DNA fragmentation

As one of the hallmarks of apoptosis, DNA fragmentation serves as a feasible tool for detection. DNA fragments (approximately 200 bp) generated by endonuclease cleavage during apoptosis can be visualized by agarose gel electrophoresis (Banfalvi, 2017). This method is favored due to its convenience. However, there are several drawbacks associated with this approach that need to be considered. For example, the sensitivity of the method is directly proportional to the number of apoptotic cells, requiring relatively a high number of apoptotic bodies in the samples. Another disadvantage is the rate of false positives that arises from the additional stress induced during the sample preparation. Additionally, the inability to detect the early stages of apoptosis constitutes yet another disadvantage of this approach (Elmore, 2007; Martinez et al., 2010).

End-labeling the resulting DNA fragments has led to the development of other derivations such as In Situ End Labeling (ISEL) and Terminal dUTP Nick End-Labeling (TUNEL). Both methods incorporate the labeled nucleotides at the site of DNA strand break either by DNA polymerase I in ISEL or terminal deoxynucleotidyl transferase in TUNEL assay. Digitoxigenin-tagged or biotinylated nucleotides used in ISEL as well as dUTP biotin nick end labeling in TUNEL can be monitored by fluorescent microscopy and flow cytometry following hybridization with appropriate probes (Ning et al., 2002; Banfalvi, 2017). The difference between the two methods relies on the location of the label on DNA. In TUNEL assay, TdT can label both the 3′- and 5′-ends as well as blunt ends, whereas in ISEL assay, labeling is performed only on the 3′-ends, which makes the former more sensitive (Hall, 1999). As both methods are based on DNA strand break detection, the rate of false positives may be relatively higher due to DNA damage rather than apoptosis.

### 2.5. Spectroscopy

The rate of apoptosis can be detected by monitoring the absorption and emission of light by the sample. This approach allows the use of different platforms ranging from microscopes and spectrophotometers, with or without fluorescent dyes, to flow cytometers based on laser excitation. DNA-fragmentation-based methods, ISEL and TUNEL, can be also categorized in this section due to the use of fluorescent-tagged nucleotides. Comet assay is another method for apoptosis detection, which is based on the analysis of DNA strand breaks (Lorenzo et al., 2013). This assay is also called single cell gel electrophoresis due to the electrophoresis of cells embedded in agarose. Following the lysis of cells with detergent and high salt, electrophoresis is carried out at high pH value, resulting in the formation of comet-like structures. The intact part of DNA migrates relatively more slowly and is observed as the head, whereas the damaged DNA migrates faster and, thus, forms the tail of the comet. This method can sense DNA stretching, nuclear budding, and apoptotic bodies containing nuclear fragments, which are the indicators of early apoptosis. The sensitivity of the method is higher and the stage-dependent detection is more likely than DNA fragmentation by agarose gel electrophoresis (Choucroun et al., 2001).

Being one of the early indicators of apoptosis, phosphatidylserine exposure serves as another useful tool to detect apoptosis. Fadok et al. (1992) reported that the macrophage phagocytosis during apoptosis in the negative selection of thymocytes is attained through the recognition of the exposed phosphatidylserine (Fadok et al., 1992). Andree et al. (1990) subsequently showed that the vascular anticoagulant α protein, thereafter, termed as annexin V, binds to the phospholipid surface on the cells in the presence of calcium ions (Andree et al., 1990). This finding was exploited by Koopman et al. (1994) to quantify the Annexin-V-positive, thus apoptotic, cells by flow cytometry via using fluorescein isothiocyanate (FITC)-labeled annexin V in the presence of a calcium buffer (Koopman et al., 1994). Flow cytometry analysis performed by fluorochrome-labeled annexin V, coupled with 7AAD or PI possessing DNA binding capacity as an indicator of late apoptosis, is now considered one of the most common methods for quantification of apoptosis (Zembruski et al., 2012).

Alterations in the mitochondrial membrane potential followed by the cytochrome c release into the cytosol are also considered an indication of early apoptosis (Martinez et al., 2010). JC-1, rhodamine 123, and its derivatives tetramethylrhodamine methyl (TMRM) and ethyl (TMRE) ester are examples of the most frequently used fluorescent lipophilic cationic probes for direct measurement of the mitochondrial potential. This process can be monitored by various methods such as flow cytometry, fluorescence-detecting microscopy, and fluorometry (Perry et al., 2011). Rhodamine-based probes exhibit aggregation-caused quenching (ACQ) characteristic (Li et al., 2020) as they accumulate at high mitochondrial membrane potential (MMP) conditions, forming aggregates and quenching fluorescence. As the MMP decreases and depolarization occurs, the fluorescent intensity increases until a complete dissociation. Apart from the monochromatic rhodamine derivatives, JC-1 acts as a dual-color probe, allowing for the rational measurement based on aggregated and monomer forms in red and green color, respectively (Perry et al., 2011). As the name denotes, JC-1 forms reversible J aggregates because of accumulation in mitochondria in a healthy state, which emits red fluorescence. In the case of apoptosis, the membrane permeability increases followed by a reduction in the electrochemical potential, resulting in a lesser uptake of JC-1, prevention of J aggregate formation and emission of green fluorescence (Sivandzade et al., 2019).

In addition to fluorescence detection, light microscopy can also be exploited to distinguish apoptotic cells via a histochemical staining procedure. Hematoxylin and eosin as well as Giemsa stainings are employed to define the morphological hallmarks of apoptosis such as dense purple chromatin and darker cytoplasm containing round bodies because of DNA fragmentation (Banfalvi, 2017).

### 2.6. Biochemical approaches

The protein components of apoptotic cascades, activated through extrinsic or intrinsic pathways, can be biochemically monitored by western blotting, flow cytometry or immunocytochemistry based on the availability of unlabeled or fluorochrome-labeled antibodies. Some of the common members of the protein network that are targeted for analysis include caspases, Bcl-2 and p53 proteins as the main regulators, cytochrome c as an indicator of the mitochondrial pathway and the truncated form of bid, t-bid, as the hallmark of the crosstalk between the intrinsic and extrinsic pathways (Ormerod, 2001). As these biochemical approaches require protein isolation or cell fixation, they are considered as end-point assays.

## 3. Noncoding RNAs

Historically, it was believed that the genetic information stored in the genome is first transcribed into messenger ribonucleic acids (mRNAs) followed by translation into proteins. This concept was embraced for a long time as proteins were considered as the structural and functional macromolecules in most, if not all, biochemical cellular processes. In fact, H19, the first functional regulatory ncRNA, was originally classified as a mRNA, which was later shown to not code for a protein (Brannan et al., 1990). Although some parts of this conventional central dogma of molecular biology still hold true, recent advances in transcriptome analyses make it imperative to revise this dogma in that only 2% of the genome codes for exons (Frith et al., 2005), requiring to account for evolutionarily maintaining such a big chunk of the genome without protein-coding capacity. Genome annotations in conjunction with the advances in RNA sequencing technologies have uncovered that the majority of the genome, without considering its coding capacity, is transcribed into some types of RNA at least once in one cell type under one condition throughout the development (Birney et al., 2007). Actually, the genome research in encyclopaedia of DNA elements (GENCODE) consortium was initially established in 2003 to map and identify primarily protein-coding genes by using comparative genome analysis (Roest Crollius et al., 2000) or gene discovery tools (Lander et al., 2001). To this extent, the presence of open reading frames, polyadenylation signals, splice sites, and promoter regions were used as the landmarks to annotate protein-coding genes, which naturally failed to annotate ncRNAs (Harrow et al., 2006). This consortium then evolved to uncover all gene variants following the realization that ncRNAs contribute greatly to the transcriptome complexity. Consequently, the consortium has identified seven major RNA biotypes, namely immunoglobulin gene (IG), processed transcripts, protein-coding transcripts, pseudogenes, read throughs, to-be-experimentally-confirmed (TEC) and T cell receptors (TR). Interestingly, the majority of these transcripts encompass non-protein-coding transcripts. As the number of and diversity in the eukaryotic proteome fails to explain the organismal complexity, these non-protein-coding transcripts may very well account for some of the organismal complexity by diversifying the eukaryotic gene regulatory mechanisms (Wilusz et al., 2009).

ncRNAs are defined as biologically important yet non-translatable transcripts that are generated through the transcription of genomic loci (Costa, 2007). It is actually quite challenging to categorize a novel transcript as a coding or noncoding RNA especially if the transcript is devoid of a long open reading frame (ORF). Perhaps, the first criterion sought in a potential ncRNA is the absence of an ORF longer than 100 amino acids (aa) (Frith et al., 2006; Dinger et al., 2008). It is noteworthy to keep in mind that some ncRNAs may possess long enough an ORF, resulting in the incorrect annotation of genes. For example, the *Xist* transcript was initially classified as a mRNA due to the presence of a 298-amino acid ORF (Borsani et al., 1991). Thus, a second criterion, the existence of a high homology in the ORF, was implemented to minimize errors (Dinger et al., 2008). Unfortunately, some ncRNA transcripts evolved from protein-coding genes and, thus, may possess a high homology to protein-coding genes. Thus, it is advised to look for experimental evidence such as in vitro translatability or polysome association in distinguishing between coding and noncoding transcripts (Galindo et al., 2007). Rarely, the experimental evidence may not be sufficient either as some genes code for both coding and noncoding RNAs through alternative splicing (Lanz et al., 1999; Kloc et al., 2005).

There appears to be a great heterogeneity in the size and shape of ncRNAs. Costa (2007) used the size heterogeneity to classify ncRNAs into 3 major subgroups: (1) long ncRNAs (300–10,000 nt), (2) small ncRNAs (30–300 nt), and (3) microRNAs (17–30 nt) (Costa, 2007). Based on their structure, however, ncRNAs can be either linear (Jorroux et al., 2017) or circular (Memczak et al., 2013). There are two types of linear ncRNAs, (1) housekeeping and (2) regulatory (Morey and Avner, 2004). Perhaps, historically, the best-known examples of linear ncRNAs are housekeeping ncRNAs such as transfer RNAs (tRNAs), ribosomal RNAs (rRNAs), small nuclear RNAs (snRNAs) and small nucleolar RNAs (snoRNAs). The recently emerging regulatory ncRNAs can be classified into five major subgroups, namely miRNAs, small interference RNAs (siRNAs), piwi-interacting RNAs (piRNAs), circRNAs, and lncRNAs (Yang et al., 2018). In this review, we will cover miRNAs, circRNAs and lncRNAs.

### 3.1. Approaches for noncoding RNA detection and quantification

#### 3.1.1. Microarray technology

Microarray technology was first employed by Patrick Brown and his team to monitor the differential expression of 45 *Arabidopsis* genes simultaneously via two-color fluorescence hybridization in 1995 (Schena et al., 1995). Potential applications of microarrays in gene expression analysis broadened the scientific horizon by facilitating high-throughput transcriptomics analyses. Prior to the microarray technology, traditional techniques such as northern blotting would be used to detect or quantify individual RNA transcripts (Alwine et al., 1977). Nowadays, microarray offers a hybridization-based, quick and powerful alternative to examine differentially expressed mRNAs and ncRNAs in the transcriptomes of various cells and tissues (Thomson et al., 2007; Yang et al., 2015; Li et al., 2019). Despite its convenience and relatively low cost, the microarray technology requires careful species- and transcript-specific probes to minimize false-positive signals. Also, it suffers from the resolution of data being lower compared to sequencing-based approaches (Table 2).

The typical workflow for a microarray experiment begins with the isolation of RNAs from an experimental sample (such as control and treated samples). Then, reverse transcription is performed to convert the template RNA into a more stable complementary DNA (cDNA) while labeling each sample with one of the two distinct fluorescent markers, for instance, Cy3 and Cy5. If the amount of RNA sample is scarce to obtain enough signal, the cDNA may be amplified, prior to fluorescent labeling, to increase the template input. The dual-fluorescent-labeled cDNA pools are then mixed and loaded into a microarray chip, which harbours thousands of probes complementary to the genes of interest. A few methods have been developed to improve the efficiency of labeling as this step appears to be highly critical. Labeling involves the use of fluorescent or biotin-labeled nucleotides that are incorporated into cDNA or cRNA during synthesis. Finally, washing, fixing, and scanning of the hybridized arrays are performed to acquire the data (Sealfon and Chu, 2011; Bumgarner, 2013).

Although the protocols are highly optimized for the analysis of mRNAs, there are additional optimization steps that need to be considered while using microarray chips for the analysis of ncRNAs. For example, microarray-based detection of miRNAs has challenges in labeling probes and hybridization due to the shorter length and lower abundance of miRNAs. Firstly, Locked Nucleic Acid-modified (LNA) capture probes may be used to eliminate acquisition problems of TM-normalised probe sets (Castoldi et al., 2006). Furthermore, control probes are helpful in improving sensitivity and accuracy during hybridization and detection (Dong et al., 2013). Normally, direct labeling is favored to eliminate PCR- and/or reverse transcription-based artificial errors. Indirect labeling might be advantageous, however, in the analysis of unstable and rare miRNAs (Castoldi et al., 2006). Secondly, oligo d(T)-based RT is not suitable for miRNA labeling since mature miRNAs lack a poly(A) tail. Several direct and indirect labeling methods were developed to circumvent this issue. RT- and PCR-based artifacts can be eliminated by using direct labeling. For instance, terminal deoxynucleotidyl transferase (TdT) and poly(A) polymerase (PAP) can be employed to add either an amine-modified or biotin-conjugated tail into the terminal hydroxyl group (-OH) of mature miRNAs to elevate sensitivity (Shingara et al., 2005; Zhao et al., 2015). Notably, T4 RNA ligase might be useful in labeling the 3′-end of miRNAs to avoid inconsistencies in the labeling efficiency that might arise from variations in the tail length (Wang et al., 2007). On the other hand, indirect labeling methods have the following advantages: 1) cDNA is more stable than RNA, therefore, more resistant to subsequent manipulations, and 2) scarce miRNAs can be labeled more efficiently (Li and Ruan, 2009). One of the best established examples is RT-based miRNA labeling through cDNA synthesis via eight-nucleotide random primer labeled with two biotin molecules (3′-(N)8-(A)12-biotin-(A)12-biotin-5′) (Liu et al., 2008). Regardless of the labeling type, the short length of miRNA may lead to cross-hybridization or background signals during hybridization steps. Thus, proper measures must be taken during chip production, with respect to the number and sequence of probes, to minimize this problem (Zhao et al., 2011; Leshkowitz et al., 2013).

Microarray-based profiling of lncRNAs and circRNAs resemble that of mRNAs. However, RNA isolation of high integrity from the control and test samples should be wellplanned. Regarding lncRNAs, rRNAs should be eliminated as a priori step to enrich the lncRNAs. Then, each sample should be amplified and transcribed into cRNAs without any 3′-bias utilizing a random priming method (Yang et al., 2015). The sample preparation for circRNAs differs slightly as treatment with RNase R is required to enrich for circRNAs prior to amplification and transcription into fluorescent circRNA (Zhang et al., 2018). It should be underlined that the random priming method is preferable for both lncRNA and circRNA quantification (Yang et al., 2015; Fan et al., 2019). Normally, microarray is an efficient tool for circRNA quantification (Li et al., 2019). However, the probes are typically designed specifically complementary to the back-splicing junction of circRNAs. Thus, it may be difficult to distinguish among the circRNA isoforms stemming from the back-splicing of the same mRNA.

#### 3.1.2. RNA sequencing technology

Over the past decade, Next-Generation Sequencing (NGS)-based transcriptome analyses have dominated the field to search for novel noncoding transcripts that might be the long-sought answer to the existence of a big chunk of a genome without any protein-coding capacity. To this extent, NGS is a widely used, indispensable and versatile method to detect and quantify ncRNAs (Anamika et al., 2016; Arrigoni et al., 2016; Handzlik et al., 2020). In addition to the use in expression profile analyses, novel ncRNA isoforms can be discovered by NGS (Arrigoni et al., 2016), attaining resolution at the nucleotide level. Thus, the major advantage of NGS, compared to microarray, is its sensitivity and resolution. Conversely, the requirement for sophisticated equipment and non-standardized data analysis tools are the main disadvantages of NGS-based profiling (Whitley et al., 2016; Corchete et al., 2020).

RNA sequencing (RNA-seq) begins with the preparation of an RNA sample of high integrity followed by rRNA depletion or poly(A)-plus transcript selection, cDNA synthesis, and library preparation (Zhao et al., 2018). The sequencing libraries are typically prepared from rRNA-depleted or poly(A)-plus-selected RNAs (Zhao et al., 2018; Shi et al., 2021). The choice depends on the biotype of RNA to be sequenced. For example, mRNA-seq libraries are constructed from poly(A)-plus-enriched transcripts as mRNAs carry a poly(A) tail (Stark et al., 2019), except for histone mRNAs. However, noncoding RNAs are not polyadenylated, making rRNA depletion a more attractive choice (Potemkin et al., 2021). In any case, rRNA depletion from total RNA is necessary to avoid contamination of the libraries with highly abundant ribosomal RNAs (Ding et al., 2018). Furthermore, circRNA-seq requires an additional step, RNase R treatment, to enrich the sample for circRNAs by eliminating linear RNAs (Sekar et al., 2019). It should be taken into consideration that the RNAse R treatment step may lead to the loss of RNAse R-sensitive circRNAs (Szabo and Salzman, 2016). In summary, a poly(A) selection protocol is recommended if the main goal is to profile protein-coding genes, whereas rRNA depletion is strongly suggested for lncRNA and circRNA differential expression analyses (Zhao et al., 2018).

Total RNA isolation followed by size-fractionation is the preferable method for miRNA enrichment prior to the library construction (Hafner et al., 2008). Reverse transcription of small RNAs followed by amplification and sequencing constitutes the basic workflow of miRNA-sequencing. Ligation and polyadenylation steps are performed to extend the short length of miRNAs, providing binding sites for primers during amplification. Notably, the error-prone nature of the miRNA-seq originates from this extension step substantially (Benesova et al., 2021).

The choice of bioinformatics analysis methods is a critical step in the analysis of RNA-seq data as it affects biological interpretation directly (Table 2). miRNA-seq, lncRNA-seq and circRNA-seq data analyses share the following four common steps: (1) data pre-processing, raw data quality control and adapter trimming (2) mapping and annotation (3) differential expression analysis and (4) functional analysis (Fu and Dong, 2018; Babarinde et al., 2019; Duan et al., 2019; Veneziano et al., 2015; Gomes-Duarte et al., 2021). Quality check of sequencing reads is necessary to remove low-quality reads. It can be performed via tools such as FastQC, FASTX-Toolkit, NGS QC Toolkit, cutadapt, and PrinSeq. Numerous RNA pre-analysis tools offer capabilities for both filtering and trimming RNA-seq data such as the NGS-QC toolkit (Liu et al., 2019). However, specific tools, like Qtrim, were developed for quality trimming (Shrestha et al., 2014). In this step, clean reads are obtained for circRNA and lncRNAs. Distinctly, the miRNA reads are trimmed and cleaned by removing the sequences smaller than 18nt or longer than 30nt (Han et al., 2020). Additionally, calculation of Q20, Q30, GC-content, and duplication level of the sequence is important to assess the quality of reads before moving onto the downstream steps, such as genome alignment (Peng et al., 2018). Typically, *de novo* assembly is more suitable while working with novel organisms, whereas the mapping assembly approach is preferable in the presence of a reference genome (Choudhuri, 2014). Lastly, differential expression analyses between control and test groups can be performed by DE analysis pipelines such as DESeq2 (Li and Wang, 2021).

For many ncRNA researchers, it is a big dilemma to select between RNA-seq and microarray as they are both powerful and comprehensive approaches that facilitate the genome-wide analysis of transcriptomes. The main difference between microarray and RNA-seq is that the former allows the profiling of known transcripts while the latter can identify novel transcripts with a wide dynamic range at a nucleotide resolution. The heightened resolution of RNA-seq permits identification of various isoforms, such as edited RNAs or template-independent extensions. The experimental cost and the availability of a reference genome are two other factors that affect the choice as well.

#### 3.1.3. Quantitative polymerase chain reaction

qPCR is a traditional nucleic acid amplification method that is widely used in ncRNA quantification (Zeka et al., 2015; Lu et al., 2018; Panda, 2018). Basically, this method relies on a direct relationship between the cycle threshold (Ct) value and the logarithm of the starting template amount (Karlen et al., 2007). The low cost makes qPCR the preferable choice to quantify differentially expressed ncRNAs compared to microarray and RNA-seq approaches. However, this approach is not high-throughput and confined to validation of known and selected candidates, without any possibility of novel transcript discovery (Yaylak and Akgül, 2022).

The short length of and high sequence similarity among miRNAs stand out as a major challenge in miRNA quantification through qPCR. Therefore, a priori size extension is employed to overcome this hurdle. The size extension of miRNAs can be attained through the use of two approaches, stem-loop RT (Chen et al., 2005) or poly(A) tailing (Shi and Chiang, 2005). The polyadenylated miRNAs can then be reverse transcribed simply by using an oligod(T) primer to generate cDNA. Subsequently, one universal and one miRNA-specific primer are used to quantitatively measure miRNA amounts.

The physical composition of lncRNAs requires the use of alternative approaches to synthesize cDNA. Random primers should be opted to synthesize cDNAs from circRNA and most lncRNAs, which lack a poly(A) tail (Wilusz, 2016; Yaylak et al. 2019). Irrespective of cDNA quality and quantity, qPCR has several disadvantages for circRNA validation and quantification. For example, divergent primers (Zhong et al., 2018) may amplify, instead of circRNAs, genomic rearrangements, trans-splicing events or cDNA generated due to template switching during reverse transcription. Genomic DNA, without any RNA, can be used as the template to examine the presence of such rearrangements (Cocquet et al., 2006; Jeck and Sharpless, 2014; Lasda and Parker, 2014). It should be noted that certain amplicons might be surprisingly long due to the presence of introns within some circRNAs. On the other hand, RNAse R treatment is a useful strategy to identify trans-splicing events typically generated from linear RNAs (Yu et al., 2014). It is very unlikely that two different RT enzymes will switch templates in the same region. Thus, candidate circRNAs may be amplified from two different reverse transcriptase-derived cDNAs to eliminate potential template-switching-event-based backsplicing junction artifacts (Lasda and Parker, 2014; Yu et al., 2014). Considering all these potential concerns, an RT-based characterization is not preferred in examining the circularity of a novel circRNA.

#### 3.1.4. Northern blotting

Northern blotting is a traditional but gold-standard method that allows detection and quantification of ncRNAs without any complex technical knowledge or sophisticated equipment (Alwine et al., 1977). Northern blotting is composed of three fundamental steps, namely the size separation of RNAs through agarose gel electrophoresis, transfer of the fractionated RNA onto a nylon membrane and hybridization-based detection and quantification (Yaylak and Akgül, 2022). Because this method involves the separation of RNAs based on their electrophoretic mobility, miRNAs of varying sizes and different types such as pre- or mature miRNAs can be simultaneously probed on the same filter (Bofill-De Ros et al., 2019; Siddika and Heinemann, 2021). Despite its simplicity and reliability, several drawbacks are associated with northern blotting. For instance, it may be challenging to distinguish among miRNAs if they share similar base composition and have the same length, whereby cross-hybridization complicates the analysis (Ouyang et al., 2019). Low sensitivity is another major disadvantage of this method, requiring the use of a high amount of RNA (He and Green, 2013). However, alternative signal detection methods have been developed to increase the specificity and sensitivity. Valoczi and his colleagues introduced LNA probes to overcome both the low sensitivity and labor problems of this technique (Válóczi et al., 2004). Furthermore, Ramkissoon developed 3′-digoxigenin-labeled RNA oligo probes as an alternative to hazardous radioactive 32_P_-labeled probes (Ramkissoon et al., 2006). Additionally, EDC [1-ethyl-3-144 (3-dimethylaminopropyl) carbodiimide] together with (DIG)-labeled LNA oligonucleotide probes not only decreases exposure time nearly 1000-fold but also increases sensitivity by generating clear signals with RNA amounts as low as 5 fmol (Kim et al., 2010).

Detection of lncRNAs by northern blotting resembles that of mRNAs. Perhaps the only point to consider is the concentration of lncRNAs being much lower compared to that of mRNAs. Analysis of circRNAs by northern blotting is performed by standard northern blotting procedures, but in combination with RNase R and RNase H treatments (Schneider et al., 2018). It is suggested that northern blotting should be preferred to validate circRNAs because it is not affected by RT-related complications (Dodbele et al., 2021). Additionally, northern blotting permits quantification of both linear template and circRNAs simultaneously.

### 3.2. microRNAs

miRNAs are an important group of endogenous single-stranded small ncRNAs of approximately 17–22 nt in length that control gene expression post-transcriptionally (Bartel, 2018). Most cognate miRNAs bind to the 3′ untranslated region (UTR) of target mRNAs although there exist miRNA recognition elements (MRE) in other parts of mRNAs as well. *lin4* and *let-7* constitute the first examples of miRNAs, which were discovered during a screen for genes that determine developmental timing in *C. elegans* (Lee et al., 1993). Actually, these miRNAs were identified as early as 1980 but remained as a mystery due to their non-protein-coding capacity (Horvitz and Sulston, 1980). The early 2000s witnessed the discovery of a number of miRNAs in *C. elegans* and *Drosophila* (Lagos-Quintana et al., 2001; Lau et al., 2001; Lee and Ambros, 2001), paving the way for searching for cloning-and sequencing-based identification of miRNAs in many other species (Pasquinelli et al., 2000; Davis-Dusenbery and Hata, 2010; Friedländer et al., 2014). Although it was difficult to distinguish between siRNAs and miRNAs at first, illumination of the biochemical basis of RNAi-based gene silencing led to the recognition of miRNAs as a new group of small ncRNAs (Mello and Conte, 2004). Doubts about the functionality of these small molecules dissipated following the observation that mutations in Dicer and AGO proteins are either fatal in animals or cause several developmental defects in animals and plants (Knight and Bass, 2001). Subsequent studies demonstrated that miRNAs play an essential role not only in development but also in key cellular processes such as metabolism, stem cell differentiation, cell death or cell cycle (Lynam-Lennon et al., 2009; Xiao and Rajewsky, 2009; Bartel, 2018).

#### 3.2.1. Biogenesis of microRNAs

**Figure 2:**
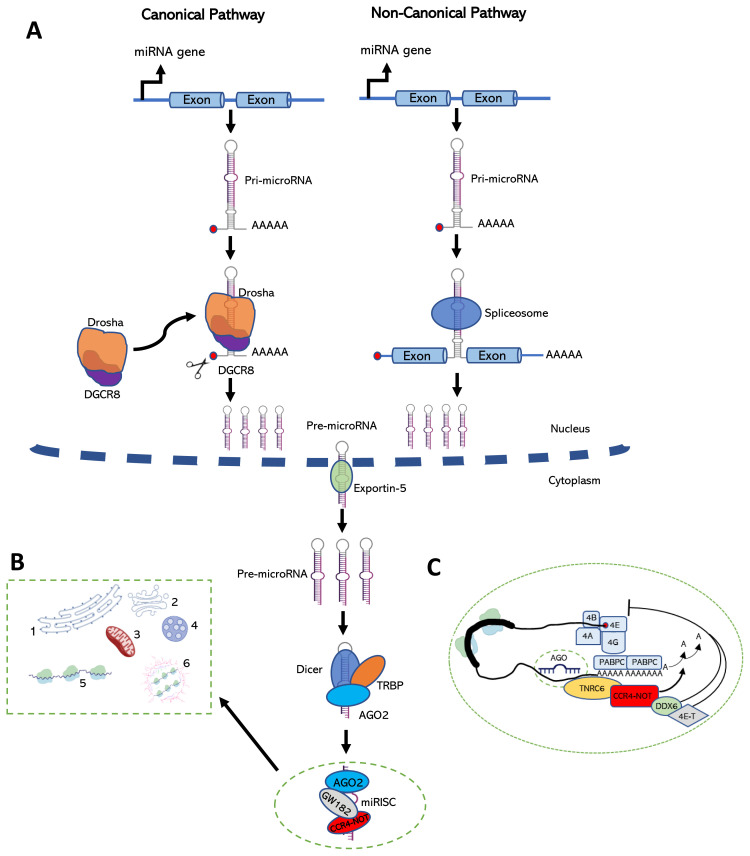
microRNA biogenesis and mode of actions. (A) Canonical and non-canonical pathways of microRNA biogenesis. In the canonical pathway, hairpin structured pri-microRNAs are converted into ~70–80 nucleotide precursor microRNAs (pre-microRNAs) by the RNase III enzyme Drosha in the nucleus. Pre-microRNAs are exported from the nucleus to the cytoplasm by Exportin-5. Dicer-processed mature microRNAs are incorporated into a microribonucleoprotein complex (miRISC complex). Non-canonical miRNA biogenesis does not involve Drosha. (B) Localization of miRISCs. miRISCs can be localized in (1) rough endoplasmic reticulum, (2) Golgi, (3) mitochondria, (4) vesicles, (5) free polysomes, and (6) cytoskeleton-bound polysomes. (C) Possible mechanisms for miRNA-mediated gene regulation. Binding of a miRNA to its target mRNA triggers either mRNA decay/cleavage or inhibition of translation.

miRNAs are produced predominantly by the canonical miRNA pathway, which is initiated by the synthesis of a primary miRNA (pri-miRNA) by RNA polymerase II (Figure 2A; Lee et al., 2002). The first processing event is carried out in the nucleus by a microprocessor complex made up of an RNA-binding protein DiGeorge Syndrome Critical Region 8 (DGCR8) and an endonuclease III enzyme, Drosha (Denli et al., 2004). The microprocessor-mediated processing in the nucleus converts the primiRNA into a 60–70-nt precursor miRNA (pre-miRNA), which is characterized by a 2-nt protruding extension in its 3′-end and a 5′-phosphate group (Lee et al., 2002). Drosha cooperates with DCRC8 in human and Pasha in *Drosophila* to perform the nuclear processing event (Denli et al., 2004; Han et al., 2004). The resulting pre-miRNAs are transported into the cytoplasm by Exportin-5, a Ran GTPase (Yi et al., 2003). The cytoplasmically-localized pre-miRNAs that possess a loop in the middle are subjected to a second round of processing by Dicer, yet another endonuclease III group of enzymes (Hutvágner et al., 2001). Dicer is accompanied with TRBP in human to generate a duplex with partial complementarity (Chendrimada et al., 2005). The polarity of the miRNA strand dictates the name of the mature miRNA. The miRNA processed from the 5′-end of the pre-miRNA hairpin is called the 5p strand, while the 3p strand stems from the 3′-end of the hairpin. Interestingly, both strands can be potentially loaded into an RNA-induced silencing complex (RISC) depending on the cell type or cellular states (Yoda et al., 2009). The strand selection at this point appears to be governed by the thermodynamic stability at the 5′-end of the miRNA duplex. The strand that is thermodynamically less stable at its 5′-end, called the guide strand, is loaded, in an ATP-dependent manner, into the RISC complex composed at least of argonaute (AGO) and a glycine and tryptophan-rich 182 protein (GW182) (Siomi and Siomi, 2009). The resulting microRNA-induced silencing complex (miRISC) typically binds to the 3′UTR of the target mRNA through impartial complementarity and represses gene expression either by inducing mRNA degradation or translational repression. In contrast to animals, plant miRNAs possess 2-O methyl groups at their 3′-ends (Yu et al., 2005). The cellular fate of the other strand of the miRNA duplex, called the passenger strand, is primarily determined by its degree of complementarity with the guide strand. For example, passenger strands without any central mismatches with the guide strands are cleaved by AGO2 (Ha and Kim, 2014).

The noncanonical miRNA biogenesis pathway involves production of pri-miRNAs that do not go through Drosha- or Dicer-mediated cleavage. Perhaps the best-known example of such miRNAs are mirtrons, those that are generated from debranched introns that enter the biogenesis process as pre-miRNAs (Ruby et al., 2007). A subset of mirtrons may possess a 5′- or 3′ tail at the premiRNA hairpin, which is processed further by a non-Drosha nuclease prior to entrance into the downstream pathway (Babiarz et al., 2008). Other than mirtrons, a second group of noncanonical miRNAs are generated from endogenous short-hairpin RNAs (shRNAs) (Babiarz et al., 2008). Endogenous versions of these shRNAs are transcribed by RNA pol II, which also determines the 5′ start site. As the 5′-end of the transcript is capped, this pathway of miRNA biogenesis yields only the 3p strand (Xie et al., 2013). Subsequent studies have shown that hairpins at the 5′ regions of certain mRNAs may serve as the Dicer substrate to produce miRNAs in a Drosha-independent pathway (Zamudio et al., 2014). A third type of non-canonical miRNAs are processed from chimeric transcripts that contain another gene in addition to the miRNA gene. In this case, an initial processing is required to release the hairpin that can serve as the Dicer substrate (Ender et al., 2008).

The miRNA processing machinery appears to be highly consistent in maintaining homogeneity at miRNA ends, accomplishing nearly 98% homogeneity in the strand selection and terminal identities (Chiang et al., 2010). However, the increase in the depth of small RNA-seq coverage revealed the existence of multiple miRNA isoforms called isomiRs that encompass heterogeneity either at a single or both ends or the final miRNA body. Such variations may have a fundamental effect on target repertoire as the isoforms end up harboring a different seed sequence especially due to the heterogeneity at the 5′ end (Bartel 2018). These isomiRs are generated through either template-dependent or –independent variations (Dhanoa et al., 2019). Template-dependent variations include trimming events carried out by exonucleases whereas template-independent post-transcriptional variations involve either RNA editing or 3′-tailing events. Most isomiRs are actually generated from miRNA duplexes, the cytoplasmic Dicer cleavage products (Dhanoa et al., 2019). Variations at the 3′-ends are produced through terminal-nucleotide-transferase or 3′-exonuclease activities (Kim et al., 2016). Such variations do not influence the target selection as they do not change the seed sequence. However, 3′-isomiRs are reported to modulate miRNA processing or stability (Ha and Kim, 2014). The production of edited miRNAs involves adenosine deaminases (ADAR, adenosine deaminases acting on RNA), which was first reported for pre-miR-22 through conventional sequencing (Luciano et al., 2004). Subsequent next-generation-sequencing (NGS) studies reported a plethora of edited miRNAs (Hoon et al., 2010). Currently, there is a free online database called IsomiR Bank (http://mcg.ustc.edu.cn/bsc/isomir/) that houses annotated isomiRs (Zhang et al., 2016).

The subcellular compartmentalization of canonical miRNAs or isomiRs is primarily determined by the composition of miRISC complexes (Figure 2B). Cytoplasmically, miRISCs can localize at different nonmembranous foci, endomembrane structures, or mitochondria (Akgül and Erdoğan, 2018; O’Brien et al., 2018). The nonmembranous compartments that were reported to serve as the cytoplasmic site of miRNA action include processing bodies (P bodies) (Pillai et al., 2005), stress granules (SGs) (Leung et al., 2006), polysomes (Olsen and Ambros, 1999), germ granules (Wu et al., 2017) and neuronal granules (Zalfa et al., 2006). Interestingly, the switches from one cytoplasmic site to another were reported for various miRNAs, and this switch appears to be governed by the constituents of the miRISC complex modulated by extracellular signals (Molotski and Soen, 2012; Wu et al., 2013; Göktaş et al., 2017; Cosacak et al., 2018). miRISCs may also function in several organelles such as the trans-Golgi network and endosomes (Bose et al., 2017), rough endoplasmic reticulum (rER) (Wu et al., 2013), multivesicular bodies (MVBs) (Gibbings et al., 2009), lysosomes (Gibbings et al., 2009), mitochondria (Kren et al., 2009) and nucleus (Gagnon et al., 2014). Additionally, numerous studies report the existence of extracellular miRNAs in biological fluids either in the form of enclosed exosomes, microvesicles and apoptotic bodies or as miRNP complexes without enclosure (Cortez et al., 2011; Turchinovich et al., 2011; O’Brien et al., 2018).

#### 3.2.2. Mode of action of miRNAs

Most miRNAs bind to the 3′ UTR of their target mRNAs but there are several studies that report the existence of miRNA response elements (MREs) on the other parts of target RNAs such as 5′-UTR, coding region or promoter regions (Bartel, 2018). Target recognition typically involves Watson-Crick pairing between the MRE and the miRNA seed region encompassing the nucleotides 2–7 (Bartel, 2009). The extent of pairing between the MRE and the miRNA dictates the repression mode. In the presence of extensive complementarity, the target mRNA destabilization is initiated through an endonucleolytic cleavage if the AGO protein has the slicing activity (Meister et al., 2004). The AGO-mediated endonucleolytic cleavage, which is the dominating mode of action in plants (Jones-Rhoades et al., 2006), has been reported only for a few mammalian transcripts (Yekta et al., 2004). In human and other mammals, a partial complementarity between the MRE and the miRNA leads to deadenylation-dependent mRNA destabilization (Figure 2C; (Jonas and Izaurralde, 2015). In this case, the AGO protein without an endonucleolytic cleavage activity recruits the TNRC6 protein, which subsequently interacts with poly(A)-binding protein (PABP) and deadenylase complexes such as the PAN2-PAN3 complex and the CCR4-NOT complex (Jonas and Izaurralde 2015). The TNRC6 protein appears to have a dual function as it also modulates the translational efficiency of the target mRNA by interacting with the CCR4-NOT complex and the DDX6 helicase (Chu and Rana, 2006). An interaction between DDX6 and eIF4E transporter then leads to translational repression by acting as a competitor for the eIF4G-eIF4E interaction, which plays an essential role in enhanced translation (Kamenska et al., 2016).

In the late 2000s, there was a dispute over whether miRNA-mediated gene regulation is primarily governed by target mRNA destabilization or translational repression in mammals (Filipowicz et al., 2008; Guo et al., 2010). Subsequent studies revealed that although translational repression also contributes to the TNRC6-mediated gene regulatory activity in animals, the ensuing target mRNA destabilization takes over the regulation as the predominant mode of regulation in post-embryonic cells (Eichhorn et al., 2014; Guo et al., 2014; Bartel, 2018). Excluding the effect of mRNA destabilization, translational repression appears to account for 6%–26% of the endogenous target repression in mammals (Eichhorn et al., 2014). Initially, conflicting results were reported with respect to the miRNA-mediated translational regulation taking place at the initiation or post-initiation stage (Nilsen, 2007; Filipowicz et al., 2008; Carthew and Sontheimer, 2009). However, a consensus has later emerged on the notion that miRNAs regulate cap-dependent translation initiation that involves ribosome scanning and eIF4A (Jonas and Izaurralde, 2015). In the embryonic cells, on the other hand, the sole repressive mode is the translational regulation as the tail shortening modulates the translational efficiency of mRNAs rather than their stability (Subtelny et al., 2014). In fact, the zebrafish embryo studies supported this notion of TNRC6-mediated translational suppression in the embryonic cells (Bazzini et al., 2012). Congruently, a considerable fraction of miRNAs was reported to associate with polysomal fractions in 1h *Drosophila* embryos, some of which switch to non-polysomal fractions upon the maternal-to-zygotic transition (Cosacak et al., 2018).

The miRNA-mediated gene regulation is not limited to target destabilization or translational repression. Interestingly, there are reports on miRNA-mediated translational activation especially under non-physiological conditions. For example, Fragile-X-mental retardation related protein 1 (FXR1) and AGO2 independently associate with mRNAs that possess AU-rich elements (AREs) in their 3′-UTRs and activate translation upon serum starvation (Vasudevan and Steitz, 2007). A similar phenomenon was reported in quiescent cells, further confirming the existence of this mode of repression under specific cellular conditions (Truesdell et al., 2012; Bukhari et al., 2016). In the nucleus, miRISCs can mediate target destabilization similar to their cytosolic effect (Nishi et al., 2013). Amazingly, miRISCs can suppress transcription, alter chromatin dynamics or modulate alternative splicing, suggesting a transcription or splicing factor-like function of miRNAs (Alló et al., 2014; Bottini et al., 2017; M et al., 2017; Miao et al., 2019).

It is important to note that miRNAs as micromanagers of post-transcriptional regulation are subject to intense genetic and epigenetic regulation as well. We refer the readers to other reviews on this topic due to space limitation (Hamid and Akgül, 2014; Gebert and MacRae, 2018; Alkan and Akgül, 2022). Additionally, recent studies suggest an intricate interplay among different types of RNAs such as mRNAs, miRNAs, and circRNAs (Rong et al., 2017; Patop et al., 2019).

#### 3.2.3. miRNAs in apoptosis

The first example of a miRNA that regulates apoptosis, bantam, was reported in *Drosophila* 18 years ago (Brennecke et al., 2003). bantam was identified during a screen for genes that modulate tissue development in *Drosophila* (Hipfner et al., 2002). It was later shown to target the proapoptotic gene hid and to suppress apoptosis during development. The same year, miR-14 emerged as the second miRNA in the fly that suppresses cell death by targeting DrICE, the *Drosophila* homologue of caspase-3 (Xu et al., 2003). The use of a library of miRNA inhibitors facilitated the functional identification of numerous pro- and antiapoptotic miRNAs in HeLa and A549 cells, clearly indicating that miRNA-mediated regulation of apoptosis is not limited to *Drosophila* (Chen et al., 2005). The ensuing years experienced an explosion of reports on miRNA-mediated regulation of apoptosis in different cellular contexts (For reviews, see (Lynam-Lennon et al., 2009; Subramanian and Steer, 2010; Chen et al., 2014; Su et al., 2015).

Bioinformatics analyses suggest that over 60% of human protein-coding genes are under selective pressure to maintain interaction with miRNAs (Friedman et al., 2009). Considering the fact that each mRNA may have multiple MREs (Chou et al., 2018) it is plausible to suggest that thousands of mRNAs may be targeted by miRNAs as part of regulation of apoptosis. In fact, there are over fifty well-documented miRNAs targeting a repertoire of pro- and antiapoptotic mRNAs involved in intrinsic or extrinsic apoptotic pathways (Figure 1 and Table 3). Strikingly, most critical regulators of apoptosis are targeted by miRNAs. To highlight a few of these miRNAs that modulate the extrinsic pathway, miR-21 was reported to regulate the FasL/Fas apoptotic pathway by directly targeting FasL in HEK293 and the pancreatic cancer PANC-1 cell line (Wang et al., 2013). The Fas receptor, on the other hand, is targeted by miR-20 in the osteosarcoma SAOS-2 cell line (Huang et al., 2012). In the intrinsic pathway, miR-204 promotes apoptosis by downregulating the expression of Bcl-2 in N87 and GTL-16 gastric cell lines in addition to HEK293T cells (Sacconi et al., 2012). The proapoptotic Bax protein is a key regulator of the intrinsic apoptotic pathway, which is regulated by miR-365 in the pancreatic cancer cell lines PANC-1 and AsPC-1 (Hamada et al., 2014). miR-27a/b modulates apoptosis by downregulating Apaf-1 in HEK293 cells and primary cortical neurons (Chen et al., 2014). Both pro- and active caspases may be targeted for regulation by miRNAs. The rate of apoptosis is attenuated in human nucleus pulposus cells by miR-155, which inhibits caspase-3 expression (Wang et al., 2011). miR-874-mediated downregulation of caspase-8 results in the induction of necrosis in cardiomyocytes (Wang et al., 2013) while miR-874 induces apoptosis in the breast cancer cell lines MCF7 and MDA-MB-231 by modulating the expression of CDK9. This suggests that the phenotypic outcome of a miRNA varies with the cellular contexts. miR-24a modulates eye morphogenesis by negatively regulating the expression of caspase-9 (Walker and Harland, 2009). There are many other regulatory genes such as STAT3, FoxO1, Akt, p53 and PI3K that are targeted by pro- or anti-apoptotic miRNAs (Figure 1 and Table 3).

### 3.3. Circular RNAs

#### 3.3.1. Characteristics of circular RNAs

circRNAs are covalently closed and functional RNA transcripts that possess neither a poly(A) tail nor a 5′-cap (Meng et al., 2017). circRNAs were first reported in viroids as early as 1976 (Sanger et al., 1976). Three years later, circRNAs were identified in the cytoplasm of eukaryotic cells by electron microscopy (Hsu and Coca-Prados, 1979). The unique RNA transcript of the DCC gene was discovered to contain two exons joined accurately at splice sites but in an inverse order relative to their genomic context. Nigro and his colleagues referred to these molecules as “scrambled exons” to describe such an abnormal RNA molecule resulting from canonical splicing (Nigro et al., 1991). These scrambled transcripts were not abundant, expressed at approximately 1/1,000th the level of the canonical DCC transcript, and mainly located in the cytoplasm. The science community disregarded these transcripts assuming that they are the products of erroneous splicing (Jeck et al., 2013). In the 1990s, the circ Sex-determining region Y (Sry) was shown to be a functional circRNA in mouse testis (Capel et al., 1993). Similarly, circMLL was observed to derive from scrambled exons (Caldas et al., 1998).

The 2010s witnessed the discovery of several circRNAs in parallel to the development of high-throughput RNA sequencing methods and appropriate bioinformatics pipelines. In 2012, Salzman and his team provided convincing evidence that circRNAs are abundant RNA isoforms in human cells (Salzman et al., 2012). Subsequently, circRNAs were shown to be evolutionary conserved across species and expressed in most organisms, such as most metazoans, archaea, yeast, and plants (Patop et al., 2019). The abundance of circRNAs is specific to cell type (Ruan et al., 2019). The circular structure of circRNAs confers resistance to exonuclease-mediated degradation by RNAse R, making them ideal biomarker candidates due to their increased stability (Ren et al., 2020).

#### 3.3.2. circRNA biogenesis and decay

Mutational analyses on circRNA expression vectors and the use of a splicing inhibitor, isoginkgetin, showed that the canonical splicing machinery is necessary and adequate for circRNA biogenesis. However, circRNAs are produced through back-splicing, which is generally coupled with canonical-splicing. There is no additional motif required for circularization other than the canonical 5′- and 3′-splice sites (Starke et al., 2015). There appears to be a global competition between back-splicing and canonical splicing in the circRNA-producing loci. The main difference between the back-splicing and the canonical splicing is that the back-splicing joins the downstream 5′-splice donor site into the upstream 3′-splice acceptor site (Lasda and Parker, 2014). This reaction is sterically highly unfavorable (Chen et al., 2020). Therefore, back-splicing is less efficient than canonical splicing; however, *cis*- and *trans*-acting elements facilitate these sterically unfavorable reactions and regulate the competition between the back-splicing and the canonical splicing (Chen and Yang, 2015). Despite the inefficiency of back-splicing, circular isoforms of hundreds of human transcripts accumulate proportional to their parental linear transcripts, probably due to their covalently-closed structure that insulate circRNAs from exonuclease-mediated degradation (Barrett and Salzman, 2016; Ma et al., 2020).

**Figure 3:**
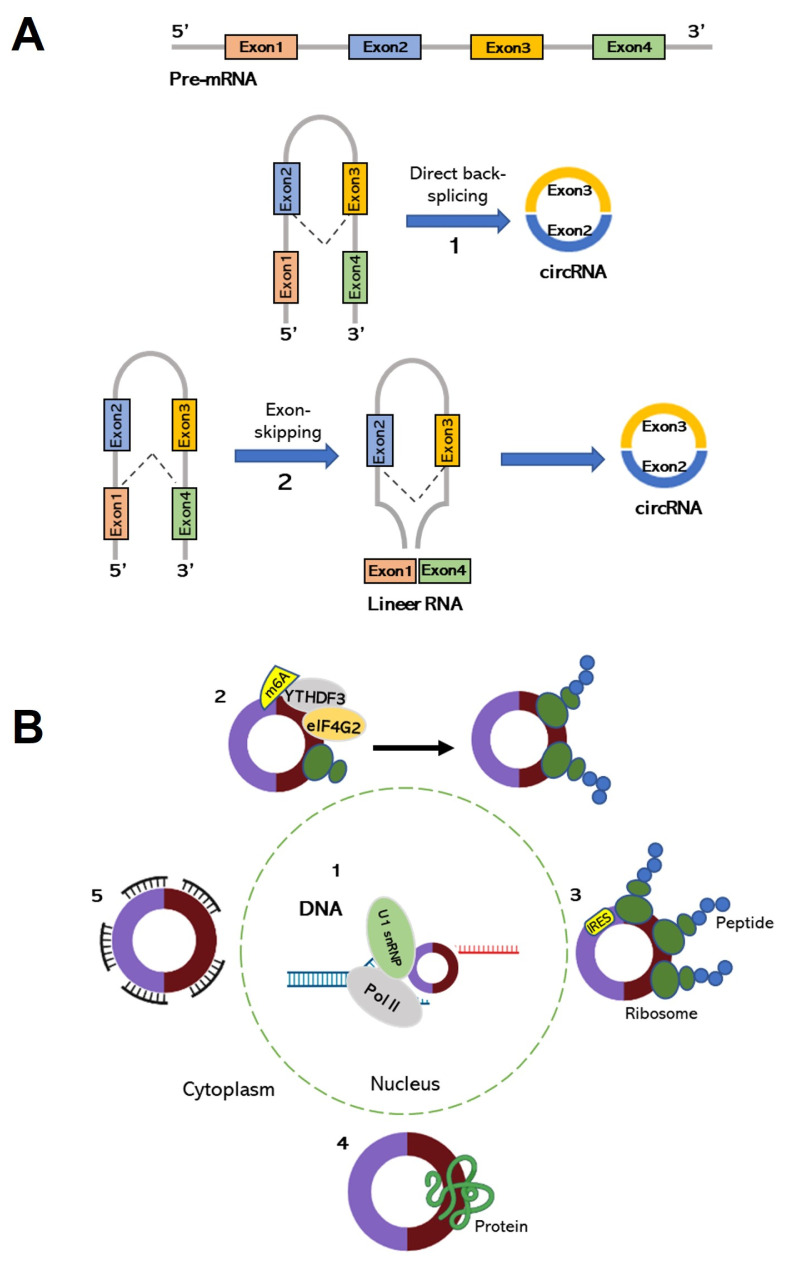
Schematic representation of circRNA biogenesis and mode of actions. (A) Two proposed models of circRNA formation. In lariat-driven circRNA biogenesis, exon-skipping leads to an alternative form of linear RNA and a lariat, which undergoes back-splicing to form a circRNA. In direct backsplicing circRNA biogenesis, flanking inverted repeats or trans-acting factor-mediated base pairing facilitates circRNA production. (B) Five modes of action of circRNAs: (1) circRNAs can promote parental gene transcription by interacting with RNA Pol II, (2) circRNAs might undergo m6A-mediated cap-independent translation, (3) circRNAs might encode unique peptides, (4) circRNAs can interact with proteins and modulate their functions, (5) circRNAs can serve as miRNA sponges to regulate the fate of target mRNA.

Two biogenesis models have been proposed to describe the underlying mechanism of the back-splicing reaction, namely lariat-driven circularization and intron-pairing circularization (Figure 3A). Broadly speaking, the fundamental difference between the two models is the order of the two modes of splicing (Chen and Yang, 2015). The subsequent back-splicing of lariats produced from exon-skipping to restore the exon content within a gene during canonical splicing produces a circRNA in the lariat-driven circularization model (Jeck et al., 2013; Yang et al., 2017). On the other hand, to overcome the natural disadvantage of the unfavorable back-splicing ligation, the inverted repeats in flanking introns bring the acceptor and donor sites into a close proximity by creating base pairing (Jeck et al., 2013; Liang and Wilusz, 2014). In some cases, specific motifs on flanking introns are occupied by RNA-binding proteins such as QKI to ease circularization (Conn et al., 2015). Surprisingly, it was documented that ds-RNA-specific adenosine deaminase (ADAR) enzymes (Ivanov et al., 2015) and ATP-dependent helicase A (DHX9) (Kristensen et al., 2018) repress the biogenesis of circRNAs by base pairing with the flanking inverted repeats. Additionally, the back-splicing may also generate isoforms from the same locus. Alternative splice site selection results in alternative back-splicing events, which generates isoforms that contain cassette exons, alternative 5′- and 3′-splice sites and introns (Li et al., 2020).

Although circRNA biogenesis is partially characterized, our current understanding of circRNA decay is highly limited. In contrast to mRNAs with poly(A) tails, circRNAs should be degraded by an endoribonucleolytic attack owing to their ring structure (Guo et al., 2020). circRNA degradation comes about through either a primary sequence-dependent- or a 3D-structure-dependent manner (Hansen et al., 2011; Guo et al., 2020). For example, circRNA Cdr1as is degraded by AGO2 protein with the assistance of miR-671 (Hansen et al., 2011). Although the parental linear mRNA and circRNA share identical primary sequences, it is unknown as to how cells distinguish between circular and linear isoforms for degradation. One potential explanation could be the unique 3D structure of circRNAs that might be recognized by RBPs to distinguish circRNA specifically.

#### 3.3.3. Functions of circRNAs

Despite the concerns over the functionality of circRNAs, increasing evidence suggests that circRNAs modulate gene expression in a cell-specific manner (Figure 3B). The biological functions of circRNAs are grouped under three mechanisms: (1) transcriptional regulation of parental linear mRNAs by exonic-intronic circRNAs, (2) miRNA sponging, and (3) gene regulation through circRNA:protein interactions (Wilusz 2018; Huang et al., 2020). Although most circRNAs are localized in the cytoplasm (Li et al., 2019), a fraction of circRNAs exist in the nucleus. circRNAs generated from exons and retained introns are classified as exon-intron circRNAs, which promote transcription of their parental genes through U1snRNP interaction (Li et al., 2015). Additionally, circular intronic RNAs (ciRNAs) can accumulate in the nucleus and induce the expression of a parental gene by regulating RNA polymerase II activity (Yang et al., 2018). Surprisingly, Xu and Zhang claim, based on the analysis of RNA-seq data from multiple tissues of humans, macaques and mice, that most mammalian circRNAs are not functional as they probably originate from splicing errors (Xu and Zhang, 2021).

Numerous studies report that circRNAs may sequester miRNAs by acting as competing endogenous RNAs or miRNA sponges (Panda, 2018). Considering the importance of miRNAs in post-transcriptional gene regulation, such circRNA:miRNA interactions may have a fundamental effect on the abundance of miRNA targets. Due to the low abundance of circRNAs, it was assumed in the mid-2010s that circRNAs should contain multiple miRNA binding sites to act as a miRNA sponge. Based on this assumption, the sponging activity of circRNAs was challenged since solely 12% of circRNAs appear to have miRNA binding sites (Ragan et al., 2019; Xu and Zhang, 2021). Additionally, other than ciRS-7, most circRNAs do not harbor more miRNA binding sites than would be expected by chance (Guo et al., 2014). Conversely, some studies documented that circRNAs with a limited number of miRNA binding sites have the ability to act as a miRNA sponge (Tang et al., 2019). Dodbele and Mutlu proposed that a scarce circRNA with a single miRNA binding site is unlikely to sponge miRNAs (Dodbele et al., 2021). In fact, the vast majority of studies about circRNA:miRNA sponging neglects the stoichiometry between miRNAs and circRNAs. For example, the use of circRNA overexpression plasmids and reporter constructs create non-physiological conditions that need to be strictly confirmed by examining endogenous miRNA:circRNA interactions (Dodbele et al., 2021).

RBPs interact with and modulate the fate of RNAs by forming ribonucleoprotein complexes. Similar to sequestration of miRNAs, circRNAs may act as protein scaffolds, decoys, sponges, and recruiters. In recent years, a plenty of circRNAs have been shown to interact with proteins through specific binding sites. Apparently, the circRNA:RBP interactions are dictated by the primary sequence information and the unique tertiary structure of circRNAs (Huang et al., 2020). The circRNA:RBP interaction is a double-edged sword. As the function of a RBP may be modulated by the circRNA, such an interaction may result in the modulation of circRNA biogenesis or function. In 2014, Ashwell-Fluss et al. reported that the excess amount of MBL protein promotes circMbl biogenesis and circMbl in turn serves as a sponge for redundant MBL proteins (Ashwal-Fluss et al., 2014). circMbl biogenesis is terminated upon the decline in the MBL protein level, and the splicing machinery switches to the production of the MBL mRNA. Multiple circRNAs can also target one protein, such as IGF2BP3, associated with 34 circRNAs (Schneider et al., 2016). Moreover, some circRNAs have been reported to encode proteins through internal ribosomal entry site-dependent and m6A-induced translation mechanisms (Prats et al., 2020). For example, circ-E-Cad encodes a unique 254 aa E-cadherin peptide, a new ligand that activates the EGFR signalling pathway (Gao et al., 2021). Of note, peptides generated by circRNA have a lot of potential as therapeutic targets and tumor biomarkers. Tumor specific nature of circRNA-encoded peptides will make them an attractive anticancer drug target (Lei et al., 2020).

#### 3.3.4. circRNAs in apoptosis

It is unquestionably vital to understand how circRNAs regulate apoptosis. Therefore, recent efforts have been geared towards the discovery and functional characterization of circRNAs that are involved in apoptosis and associated diseases such as cancer, neurodegenerative diseases, and cardiovascular diseases (Lee et al., 2019). One of the first examples of apoptosis-regulating circRNA, hsa_circ_000595, was reported in 2015 (Zheng et al., 2015). Hsa_circ_000595 is significantly upregulated under hypoxic conditions and its knockdown attenuates hypoxia-induced apoptosis of human aortic muscle cells. A hsa_circ_000595:miR-19a regulatory axis appears to be involved in this process. CiRS-7 is one of the well-characterized circRNAs with more than 70 conventional binding sites for miR-7 (Hansen et al., 2013). Overexpression of ciRS-7 increases caspase-3 activity and the rate of apoptosis in cardiomyocytes. Mechanistic investigations demonstrated that Cdr1as acts as a miR-7a sponge by inhibiting the regulation of miR-7a on its targets PARP1 and SP1. Nevertheless, PARP and SP1 were invincibly upregulated and, myocardial infarction development was elevated through ciRS-7 overexpression with aggravated cardiac infarct size (Geng et al., 2016). Few months later, circANRIL was reported as a regulator of rRNA maturation process and atherosclerosis in human (Holdt et al., 2016). circANRIL activates p53 and triggers apoptosis by impairing rRNA biogenesis. In particular, circANRIL binds the carboxy terminal domain of pescadillo homologue 1 (PES1) protein. Entrapment of PES1 by circANRIL disrupts rRNA:PES1 interaction, resulting in impaired rRNA biogenesis (Holdt et al., 2016). circRAR1 was reported as a positive regulator of lead-induced neurotoxicity through interacting with anti-apoptotic miR-671, which targets caspase-8 and p38 (Nan et al., 2017). Besides, a positive regulatory relationship between circRAR1 and lncRNA RPA is documented as one of the first examples of a regulatory interaction that modulates neural apoptosis through a circRNA-lncRNA-miRNA axis (Nan et al., 2017). circFOXO3, which is a proapoptotic circRNA (Du et al., 2017), has a strong tendency to bind MDM2, resulting in the release of FOXO3 from MDM2. The released FOXO3 promotes apoptosis by upregulating Puma expression while MDM2:p53 interactions modulate polyubiquitination of p53 (Du et al., 2017).

There are 22 publicly available and comprehensive circRNA databases that are maintained up-to-date (Tang et al., 2020). Three of those, circBase, Circ2traits, and circFunBase, are widely used in the circRNA field. LncDisease, circFunBase, and CircR2 are the databases that house the experimentally validated circRNAs. Table 4 summarizes the circRNAs that are associated with apoptosis.

### 3.4. Long noncoding RNAs

#### 3.4.1. Characteristics of long noncoding RNAs

**Figure 4:**
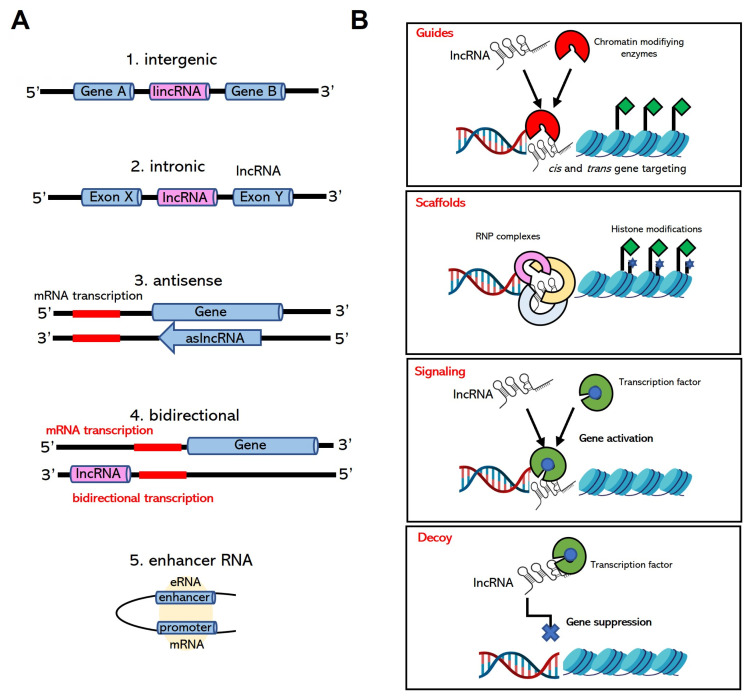
lncRNA classification and mode of action. (A) lncRNA classification. lncRNAs are classified, based on their genomic position, as intergenic, intronic, antisense, bidirectional and enhancer RNA. Intergenic lncRNAs are localized between two protein coding genes and transcribed from intergenic regions, while intronic lncRNAs are processed from introns of protein coding genes. Antisense lncRNAs are transcribed from the complementary strand of a protein coding gene. Bidirectional lncRNAs are transcribed from both stands, and enhancer RNAs are transcribed from enhancer regions. (B) lncRNA mode of action. lncRNAs act as molecular guides, scaffolds, signals, and decoys in cell. lncRNAs mediate the recruitment of chromatin modifiers to modulate gene expression of target gene expression and chromatin dynamics as molecular guides. lncRNAs can direct the assembly of protein complexes to target genes as molecular scaffolds. lncRNAs are transcribed as response to specific stimuli and work in a cooperation with transcription factors to regulate transcription of downstream genes as molecular signals. lncRNAs function as molecular decoys by blocking transcriptional regulators.

lncRNAs are a class of ncRNAs longer than 300 nucleotides in length and lack the ability to encode a protein (Costa, 2007). Similar to mRNAs, lncRNAs are transcribed by RNA polymerase II and may contain a 5′-cap and a poly(A) tail. Although having similar biochemical properties with mRNAs, lncRNAs exhibit low expression levels in the cell and are relatively poorly conserved (Statello et al., 2020). Based on their genomic position, lncRNAs can be classified as antisense (overlaps with antisense strand of protein coding gene), intronic (transcribed from intron of protein coding gene), bidirectional (bidirectional transcription of protein coding gene), intergenic lncRNAs (localized between two protein coding genes) and enhancer RNAs (eRNAs) (Fernandes et al., 2019) (Figure 4A).

The science community primarily focused on studies on the differential expression of protein-coding genes in the early 1980s. At the end of the decade, the findings on genomic imprinting were embraced with great excitement and led to the discovery of the first lncRNA *H19* (Bartolomei et al., 1991). Transcribed by RNA pol II with a small open reading frame and a poly(A) tail, H19 was initially categorized as a mRNA. Concurrently, the cytoplasmically localized *H19* was shown not to be associated with the translational machinery (Brannan et al., 1990), priming the initiatives to explore the existence of other lncRNAs with biological functions. Indeed, the discovery of *Xist*, and its functions on silencing the whole X chromosome, were a pioneering study for the identification and characterization of lncRNAs that emerged in 2000s upon the completion of the Human Genome Project (HGP) (Penny et al., 1996). Further studies have shown that lncRNAs originate from transposable element exaptation or duplication of already existing lncRNAs. Additionally, some lncRNAs may stem from pseudogenization of protein coding genes due to deleterious mutations, which results in the loss of their translation ability (Marques and Ponting, 2014).

Earlier studies have questioned the functionality of lncRNAs, arguing that lncRNAs could be transcriptional noise (Costa 2007). However subsequent studies have shown that they are evolutionarily conserved (Necsulea et al., 2014). Although the extent of conservation among lncRNAs is not quite to that of mRNAs, the promoter regions of lncRNAs are particularly conserved (Pang et al., 2006). Additionally, it is reported that transposable elements can contribute new sequence elements to the conserved lncRNAs throughout the evolutionary process (Hezroni et al., 2015). According to the up-to-date annotation of the GENCODE Project, the human genome consists of 60,880 genes including 17,957 lncRNAs and 19,954 protein coding genes (Frankish et al., 2021). The number of annotated genes may increase through the discoveries of new features and extension of annotation of alternatively spliced transcripts of lncRNAs and protein coding genes.

#### 3.4.2. Biogenesis of lncRNAs

Even though the global features of lncRNAs and mRNAs suggest they have similar biochemical properties in common, lncRNAs contain fewer and longer exons than mRNAs. Additionally, they tend to localize primarily in the nucleus and show lower expression patterns (Hezroni et al., 2015). Recent studies have shown that the relatively less abundance of lncRNAs is related to their mode of transcription. Since the lncRNA gene promoters harbor repressive histone modifications, they present a low expression pattern (Lagarde et al., 2017). Apparently, lincRNAs have relatively weaker co-transcriptional splicing and poly(A) signal-independent Pol II termination. This situation is followed up by accumulation of lncRNAs on chromatin and their degradation by RNA exosome. The functional lincRNAs mostly escape from the nuclear surveillance process to accumulate at specific cell types (Schlackow et al., 2017).

In contrast to the lincRNAs targeted by nuclear surveillance process, chromatin-tethered lncRNAs may comprise a specifically high number of U1 small nuclear RNA binding sites (Yin et al., 2020). U1 small nuclear ribonucleoprotein interacts with RNA polymerase II and binds to chromatin at specific loci that leads to accumulation of chromatin-tethered lncRNAs. Additionally, accumulated lncRNAs sequester the action of Pol II-associated elongation factor SPT6 (Yin et al., 2020). Additionally, weak-splicing profile of lncRNAs is associated with having long distance between branch point and 3′ splice site and weak internal splicing signals (Melé et al., 2017).

lncRNAs are found dispersed through nucleus, nucleolus, cytoplasm, and mitochondria. The enrichment of lncRNAs at specific cellular compartments may determine their mode of action. RNA-seq analysis of various human tissues showed the enrichment of lncRNAs in the nucleus, specifically associated with chromatin in several cell lines (Derrien et al., 2012). Subsequent microarray analysis of K562 human myelogenous leukemia cell line demonstrated the localization of fifty-four percent of expressed lncRNAs in the cytoplasm. The majority of lncRNAs in the cytoplasm are found in polysome fractions and play a role in the control of mRNA stability and translation (Carlevaro-Fita et al., 2016). PYCARD-AS1 is a quintessence of lncRNAs which function at different subcellular localizations with specific functions. PYCARD-AS1 is an antisense lncRNA to PYCARD, a pro-apoptotic gene (Miao et al., 2019). PYCARD contains N-terminal PYD domain and C-terminal Caspase recruitment domain (CARD). PYCARD-AS1 exerts functions in regulation of intrinsic and extrinsic pathways of apoptosis through localizing in the nucleus or cytoplasm (Ohtsuka et al., 2004). PYCARD-AS1 inhibits transcription of PYCARD via epigenetic regulation and inhibits PYCARD-regulated apoptosis in nuclei while PYCARD-AS1 interferes with PYCARD mRNA and prevents its translation in the cytoplasm (Miao et al., 2019).

#### 3.4.3. Mode of action of lncRNAs

lncRNAs can be classified based on their functions as molecular signals, decoys, guides, and scaffolds (Gao et al., 2020). lncRNAs, which are transcribed in response to a specific stimulus, are described as molecular signals (Figure 4B). This type of lncRNAs may modulate the transcription of downstream genes themselves or in conjunction with transcription factors. One of the well-known examples of a molecular signal is lincRNA-p21, which is regulated by p53 upon DNA damage. The binding of p53 to the CDKN1A locus activates the transcription of CDKN1A and some other lncRNAs, lincRNA-p21 and PANDA. lincRNA-p21 mediates the p53-dependent gene repression by recruiting heterogeneous nuclear ribonucleoprotein K (hnRNP-K), and this interaction triggers p53-dependent apoptosis (Hung et al., 2011).

Certain lncRNAs act as decoys by directly binding the transcriptional regulators and impairing their functions or hindering the binding of transcriptional factors to the genomic DNA (Balas and Johnson, 2018) (Figure 4B). For example, NF-YA, a nuclear transcription factor, activates apoptosis-related genes. p53-induced PANDA lncRNA in turn binds to NF-YA and suppresses the expression of proapoptotic genes such as CCNB1, FAS, PUMA and NOXA (Hung et al., 2011). Additionally, lncRNAs may serve as endogenous competitors of RNAs (ceRNAs) or miRNA sponges as part of post-transcriptional gene regulation. As stated previously, miRNAs bind to the 3′ UTR of target mRNAs and micromanage their expression. lncRNAs compete with mRNAs for binding to miRNAs, resulting in interference with miRNA:mRNA interactions (Alkan and Akgül, 2022). lncRNA:miRNA sponge mechanism also can be classified as decoy mechanism. For example, OIP5-AS1 lncRNA sponges miR-216a-5p, resulting in derepression of the GLO1 mRNA (Wu et al., 2021).

Genome structure and organization is achieved by an intricate folding of the genomic DNA mediated by numerous DNA:protein interactions. As a third mechanism, lncRNAs can act as guiding molecules to recruit chromatin modifiers, ribonucleoprotein complexes and transcription factors to the target sites to modulate chromatin dynamics and gene expression (Wang and Chang, 2011) (Figure 4B). lncRNA HOTAIR is one of the typical examples of guide lncRNAs. The two ends of lncRNA HOTAIR interact with different histone modification complexes and promote histone methylation/demethylation of target genes. The 5′-end domains of HOTAIR interacts with Polycomb repressive complex 2 (PRC2). This interaction augments histone H3 lysine 27 methylation (Gupta et al., 2010). On the other side, the 3′-end domain of lncRNA HOTAIR binds LSD1/CoREST/REST complex and, this interaction promotes demethylation at H3 lysine 4 residues (Tsai et al., 2010). In addition to acting as guide molecules, lncRNAs may also mediate the assembly of protein complexes by serving as scaffolding molecules (He et al., 2019) (Figure 4B). A study in human aortic smooth muscle cells (HASMC) showed that the ANRIL lncRNA acts as a molecular scaffold for the binding of WDR5 and HDAC3 proteins. The resulting WDR5/HDAC3 complex activates the transcription of NOX1 (NADPH oxidase) by deacetylation (Zhang et al., 2020).

By using the molecular mechanisms, lncRNAs modulate gene expression at epigenetic, transcriptional, and post-transcriptional levels (Gao et al., 2020). lncRNAs take part in epigenetic mechanisms by interacting with chromatinmodifying enzymes and remodelling complexes. As such, lncRNAs can either promote or prevent the recruitment of chromatin modifiers/remodelers (Zhang et al., 2019). For example, while *Xist* promotes the recruitment of components of PRC2 and induces heterochromatin formation by H3K27me3 deposition, lncPRESS1 interacts with SIRT6, a histone deacetyltransferase, and inhibits SIRT6 function, thereby causing activation of transcription at pluripotency gene promoters (Jain et al., 2016). The interaction of lncRNAs with DNA methyltransferases (DNMT) changes gene expression at chromatin loci. As an example, DBCCR1-003 lncRNA binds to DNMT1 and blocks the methylation of DBCCR1 by DNMT1 in bladder cancer cells (Qi et al., 2016). Furthermore, lncRNAs can regulate gene expression transcriptionally by recruiting or sequestering the regulatory protein complexes in cis- or in trans. To this extent, lncRNAs may directly interact with transcription factors and can change gene expression. For instance, MALAT1 recruits the Sp1 transcription factor to the LTBP3 promoter to activate the transcription of LTBP3 (Li et al., 2014). Lastly, lncRNAs may interact with RBPs to regulate gene expression post-transcriptionally. As such, mRNA stability, translation, splicing and subcellular localization may be modulated by lncRNAs (He et al., 2019). For example, the lncRNA pTENpg1 post-transcriptionally regulates the tumour suppressor gene PTEN, which plays an essential role in the maintenance of cell homeostasis as a negative regulator of the PI3K-Akt pathway (Xing 2010). PTENpg1 regulates PTEN by sequestering the miRNA that binds to the 3′ UTR of PTEN (Poliseno et al., 2010).

#### 3.4.5. lncRNAs in apoptosis

lncRNAs may regulate apoptosis by modulating the activity of transcription factors, histone modification complexes and miRNAs or by altering the stability of apoptosis-related proteins (Takeiwa et al., 2021). Initial efforts were geared towards uncovering lncRNAs that modulate apoptosis and/or proliferation in cancer cells. For example, linc-p21 was documented to activate p53 and apoptosis upon DNA damage (Huarte et al., 2010). The SPRY4-IY1 lncRNA was reported to regulate apoptosis in the LOX-IMX1 melanoma cells (Khaitan et al., 2011). The lnc-CCNL1-3:1 lncRNA is upregulated in polycystic ovary syndrome (PCOS) patients. The use of a RPIseq algorithm-based prediction tool suggested a potential interaction between FOXO1 and lnc-CCNL1-3:1 (Huang et al., 2021). Rescue experiments showed that knockdown of FOXO1 attenuates the effects of CCNL overexpression on cell apoptosis (Huang et al., 2021). Apparently, a specific interaction between this lncRNA and FOXO1 is essential for the nuclear retention of FOXO1 and corresponding apoptosis. In another study, a transcriptomics profiling study showed the expression of as many as 10,214 differently expressed lncRNAs under apoptotic conditions induced by cisplatin (Gurer et al., 2021). These studies suggest that lncRNAs may modulate apoptosis in a pathway-dependent manner.

lncRNAs may regulate the intrinsic pathway of apoptosis by activating p53 or regulating the downstream target genes of intrinsic pathways such as Bcl-2 family members (Shi et al., 2018; Wu et al., 2020). Taurine up regulated 1 lncRNA (TUG1) modulates apoptosis by regulating different target genes in different cell types (Liu et al., 2017; Long et al., 2018). A study reported that a direct interaction between EZH2 and TUG1 alleviates the expression level of pro-apoptotic BAX in lung cancer cells (Liu et al., 2017). Another study showed the oncogenic role of TUG1 by enhancing the astrocyte elevated gene-1 (AEG1) expression by sponging miR-129-5p in human malignant melanoma. Activation of this regulatory axis leads to an increase in the rate of apoptosis through downregulation of Bcl-2 and upregulation of cleaved caspase -3 in TUG1-suppressed melanoma cells (Long et al., 2018).

Urothelial cancer associated 1 (UCA1) is a lncRNA upregulated in bladder cancer cells. Studies showed that binding of Ets-2, a transcription factor that binds to the UCA1 promoter, modulates its transcription (Wu et al., 2013). Ets-2 transcriptionally regulates UCA1, resulting in activation of the Akt signalling pathway and suppresses the bladder cancer cell apoptosis (Wu et al., 2013). Despite the presence of high levels of endoplasmic reticulum (ER) stress in cancer cells, the mechanism of how cancer cells evade apoptosis under these conditions is not elucidated completely (Rozpedek et al., 2016). It was shown that golgin A2 pseudogene 10 (GOLGA2P10) lncRNA protects hepatocellular carcinoma cancer (HCC) cells from ER-stress-induced apoptosis by modulating the intrinsic pathway. An increase in transcription of GOLGA2P10 via ER-stress inducers leads to the induction of PERK/ATF4/CHOP signalling pathway. Mechanistically, GOLGA2P10 interferes with the ER-stress-induced apoptosis by modulating BCL-xL and phosphorylated-BAD levels in HCC cells (Wu et al., 2020).

Regulation of the extrinsic apoptotic pathway can be mediated directly or indirectly by lncRNAs (Jiang et al., 2021). For example, alternatively spliced soluble Fas receptor (sFas) that exclude exon 6 leads to inhibition of apoptosis by interfering Fas ligand (FasL) (Bonnal et al., 2008). The regulation of alternative splicing of Fas is achieved by lncRNA antisense transcript of Fas (FAS-AS1) in lymphomas. FAS-AS1 binding to RBM5 inhibits the exon 6 skipping in alternative splicing mediated by RBM5 and production of sFAS. Subsequent studies showed that hyper-methylation of FAS-AS1 by EZH2 causes the production of sFas in lymphomas and sequestration of apoptosis by sFas (Sehgal et al., 2014). HOX antisense lincRNA HOXA-AS2 is upregulated upon ATRA (all trans retinoic acid) treatment in NB4 cell line (Zhao et al., 2013). ATRA-treated HOXA-AS1 knockdown cells exhibited enhanced caspase-3, 8, -9 activities and BAX levels. Subsequent studies uncovered that TRAIL levels were upregulated, and application of TRAIL-neutralizing antibody partially suppressed caspase-8 and -9 activities in ATRA-treated HOXA-AS1 knockdown cells. Overall, HOXA-AS1 acts as a repressor of apoptosis and TRAIL may be partially involved in this regulatory mechanism in ATRA-treated NB4 promyelocytic leukemia cells (Zhao et al., 2013). Despite the potential use of TRAIL as a chemotherapeutic target, recent reports show the emergence of resistance of pancreatic cancer cells to TRAIL-induced apoptosis (James and Griffith, 2014). HOTAIR is expressed at low levels in cells sensitive to the TRAIL-induced apoptosis while it exhibits high expression level in the resistant cells. Apparently, the sensitivity of pancreatic cells is correlated with the HOTAIR expression level (Yang et al., 2017). HOTAIR overexpression results in the inhibition of death receptor 5 (DR5) by EZH2-mediated trimethylation of the lysine residue of histone 3 around the DR5 promoter (Yang et al., 2017).

lncRNAs may target tumour suppressor genes such as p53 and PTEN to control apoptosis (Levine and Oren, 2009; Yamada and Araki, 2001). For example, lncRNAGas5 binds to miR-103 and sequesters it away from PTEN. This interaction enhances the expression of PTEN and promotes apoptosis in endometrial cancer cells (Guo et al., 2015). On the other hand, if lncRNA-GAS5 binds to the DNA binding domain of glucocorticoid receptor (GR), it blocks the interaction between GR and glucocorticoid response element (GRE). By serving as a decoy for GRE, lncRNA-GAS5 blocks the transcription of GR and neutralizes the anti-apoptotic effects of GR, sensitizing cells to apoptosis (Kino et al., 2010). MEG3 activates p53 by downregulating its negative regulator, MDM2. The underlying mechanism of downregulation of MDM2 expression by MEG3 is not clear yet (Zhou et al., 2007). With respect to apoptosis, transactivation of p53 by MEG3 increases caspase 3 expression levels and decreases Bcl-2 and Cyclin D1. MEG3 promotes apoptosis and inhibits proliferation by activation of p53 in human osteosarcoma cells (Shi et al., 2018).

#### 3.4.6. lncRNA databases

Advances in transcriptome-wide studies have led to the establishment of lncRNA databases to maintain the resulting massive data (Fritah et al., 2014). The most widely used databases include LNCipedia (Volders et al., 2019), LncDisease (Bao et al., 2019) and lncRNAdb (Amaral et al., 2011) as they house the experimentally validated interaction of lncRNAs with other macromolecules. Databases such as DIANA-LncBase (Wang et al., 2016) and TargetScan (Agarwal et al., 2015) may be used to predict putative lncRNA-miRNA interactions. Based on the lncRNA-Disease database, we listed the lncRNAs with experimentally validated functions in apoptosis of different cancer cells in Table 5 (Bao et al., 2019).

## 4. Conclusion

Apoptosis is a complex cellular mechanism that plays a critical role in the maintenance of organismal homeostasis throughout development. Undoubtedly, apoptosis is regulated by epigenetic, transcriptional and post-transcriptional gene regulatory mechanisms mediated by protein coding genes. The accumulating evidence demonstrates that these regulatory proteins are regulated by a diverse biotype of ncRNAs, ranging from miRNAs to lncRNAs and circRNAs. It is important to underline the existence of other types of ncRNAs, such as piRNAs or tRNA-derived fragments (tRF) that are known to be involved in apoptotic processes (Cosacak et al., 2018; Zeng et al., 2020). More excitingly, the emerging studies document an intricate interplay among various ncRNAs (Patop et al., 2019; Rong et al., 2017). For example, a circRNA may serve as a sponge for a miRNA, which may be regulating the stability of a target lncRNA that potentially interacts with a DNA-binding protein. Thus, it will be highly informative to uncover this complex and dynamic network of interactions between ncRNAs and other macromolecules. Additionally, a recent report on small ncRNAs modified with N-glycans and displayed on the surface of living cells opens a new avenue, expanding the role of ncRNAs in extracellular biology (Flynn et al., 2021).

## Figures and Tables

**Table 1:** Apoptosis detection methods.

Method	Platform	Advantages	Disadvantages
Scanning electron microscopy (SEM)	- Electron microscopy	- Provides information from a larger area on the surface	- Lower resolution compared to TEM - Requires expertise for using the instrument - Inaccuracy due to variations of apoptotic pattern among cell types
Transmission electron microscopy (TEM)	- Electron microscopy	- Higher resolution than SEM - More detailed information of the inner structure is obtained	- Data is obtained from a smaller area than SEM - Specimen preparation is labor intensive - Specimen exposed to harsh processing conditions may destroy - Requires expertise for using the instrument - Inaccuracy due to variations of apoptotic pattern among cell types
DNA fragmentation	- Agarose gel electrophoresis	- Convenient to perform	- Sensitivity is proportional to number of apoptotic cells - False positivity due to additional stress during sample preparation - Inability to detect early phases of apoptosis
In situ end labeling (ISEL)	- Fluorescence microscopy - Flow cytometry	- N.A.	- Less sensitive than TUNEL due to labeling only on 3′- ends - False positive results due to DNA damage other than apoptosis
Terminal dUTP Nick End-Labeling (TUNEL)	- Fluorescence microscopy - Flow cytometry	- TdT can label both the 3′- and 5′-ends and blunt ends, more sensitive than ISEL	- Expensive due to enzyme and probes - False positive results due to DNA damage other than apoptosis
Comet assay	- Fluorescence microscopy	- Stage-dependent detection is possible - Sensitivity is higher than DNA fragmentation	- Genotoxicity and cytotoxicity can lead to false positive results - Variability in case of manual scoring
Annexin V staining	Flow cytometry	- Detection in early apoptosis - Direct quantification is possible by flow cytometry	- Should be assayed subsequent to staining, cannot be stored for later detection
Mitochondrial membrane potential measurement	- Fluorescence microscopy - Flow cytometry - Confocal microscopy – Fluorimetry	- Can be performed either by live or fixed cells	- Indirect method for assessing apoptosis - Not applicable for all pathways
Hematoxylin-eosin and Giemsa staining	- Light microscopy	- Cost-effective	- Detects later events of apoptosis - May underestimate the rate of apoptosis therefore should be combined with another method
Protein-based assays	- Western-blotting - Fluorescence microscopy	- Detection can be performed in a mechanistic approach	- Time-consuming - Difficulty in troubleshooting due to multistep nature - Requires additional procedures like fixation or isolation

**Table 2:** Comparision of ncRNA analysis methods.

Method	Sensitivity and Specificity	Advantages	Disadvantages	High–throughput	Quantitativeness
**Microarray**	- Less specific and less sensitive than NGS and qPCR	- Cheaper than RNA-seq - Easier data processing than RNA-seq - Fast high throughput profiling	- Cannot distinguish alternative splicing events - Low sensitivity for detecting rare transcripts	Yes	Quantitative
**Next Generation Sequencing**	- Very specific - Adjustable sensitivity	- Detection of isoforms - Profiling of organisms without a reference genome - Broader dynamic range than microarray, adjustable sequencing depth	- Massive data, difficult to store - Bioinformatic expertise required - Non-standardized data analysis tools affect biological interpretation	Yes	Quantitative
**Quantitative Real Time PCR**	- Sensitive - Medium specificity	- Easy to perform - Cheap	- To quantify known ncRNAs - Challenges in primer designs - Artifacts due to genomic rearrangement, trans splicing and template switching (circRNA) - Amplification problems due to short length of miRNA	Medium	Quantitative
**Northern Blotting**	- Low sensitivity - High specificity	- Ability to detect all forms of a transcript (circRNA– linear vs circular isoforms) - RNase H based validation of circularity (circRNA) – Cheap - Reverse transcriptase independent	- Time consuming - Validate known ncRNAs - Hazardous if performed with radioactive isotopes - High amount of starting material	No	Semi-quantitative

**Table 3:** MicroRNAs in apoptosis.

Disease	miRNA	Tissue/Cell line	Regulation	Targeted member	References
Acute kidney injury	let-7/miR-98 family	HUVECs	Up	Caspase-3	(Li et al., 2015)
Acute Myeloid Leukemia Cells	hsa-miR-34a	HL-60 and THP-1 cells	Down	HMGB1	(Liu et al., 2017)
Alcoholic liver disease	hsa-miR-21	Hepatic stellate cells, PANC-1 cells	Up	FAS	(Francis et al., 2014; Wang et al., 2013)
Breast cancer	hsa-miR-301	MCF-7, MDA-MB-231, MDA-MB-468, and T47D	Up	PTEN, FOXF2, and	(Shi et al., 2011)
Breast cancer	hsa-miR-99a	SK-BR-3, MDA-MB-435s, MCF-7 and MDA-MB-231 cells	Down	mTOR	(Hu et al., 2014)
Breast cancer	hsa-miR-101	MCF-7, MDA-MB-231 cells	Down	Jak2	(Wang et al., 2014)
Breast cancer	hsa-miR-142-3p	Breast cancer and normal breast tissues	Up	Bach-1	(Mansoori et al., 2019)
Breast cancer	hsa-miR-192	Hs578Bst, MDA-MB-231, MCF-7	Down	Caveolin 1	(Chen et al., 2019)
Breast cancer and normal tissues	hsa-miR-874	MCF7 and MDA-MB-23	Up	CDK9	(Wang et al., 2013)
Cardiomyocytes	hsa-miR-874	Cardiomyocytes	Down	Caspase-8	(Wang et al., 2013)
Cervical cancer	hsa-miR-744	HeLa, C-4-1, Ca Ski, SiHa	Up	BCL 2	(Chen and Liu 2016)
Cervical cancer	hsa-miR-143	HeLa cells	Up	HIF-1α	(Zhao et al., 2017)
Cervical cancer, Prostate cancer	hsa-miR-133b	HeLa cells, PC-3 cells	Up	FAIM	(Coccia et al., 2020)
Cholangiocarcinoma	hsa-miR-29	KMCH cells	Down	Mcl-1	(Mott et al., 2007)
Chronic lymphocytic leukemia	hsa-miR-15a	Peripheral blood	Down	BCL2	(Cimmino et al., 2005)
Chronic lymphocytic leukemia	hsa-miR-16-1	Peripheral blood	Down	BCL2	(Cimmino et al., 2005)
Colorectal cancer	hsa-miR-30b	W480, DLD1, HCT116, HCT15, KM12 and Ls174t cells	Down	KRAS, PIK3CD and BCL2	(Liao et al., 2014)
Colorectal cancer	hsa-miR-148a	RKO, SW480 and Lovo	Down	Bcl-2	(Zhang et al., 2011)
Epithelial ovarian cancer	hsa-miR-124	SKOV3 and OCVAR3 cells	Down	PDCD6	(Yuan et al., 2017)
Esophageal squamous cell carcinoma	hsa-miR-141	KYSE cell lines	Up	YAP1	(Imanaka et al., 2011)
Gastric cancer	hsa-miR-18a	MGC-803, HGC-27	Down	HIF 1 ALPHA	(Wu et al., 2015)
Gastric cancer	hsa-miR-421	MGC-803, HGC-27, BGC-823, SGC-7901	Up	CASPASE 3	(Wu et al., 2014)
Gastric cancer	hsa-miR-106a	Stomach	Up	FAS	(Wang et al., 2013)
Gastric cancer	hsa-miR-204	N87, GTL-17, HEK293T	Down	BCL2	(Sacconi et al., 2012)
Gastric cancer	hsa-miR-136	MGC-802, BGC-823, SGC-7901, AGS, SNU-1, SNU-16, and RF-1 and GES-1 cell lines	Up	AEG-1, BCL2	(Yu et al., 2018)
Gastric cancer	hsa-miR-185	MGC803	Up	Bcl-2, surviving and XIAP	(Fan et al., 2018)
Gastric cancer	hsa-miR-200c	BGC-823	Down	EDNRA	(Wei et al., 2018)
Gastric cancer	hsa-miR-100	MGC-803, HGC-27 and MKN-45 cells	Up	RNF144B	(Yang et al., 2016)
Glioma	hsa-miR-125a-3p	Normal human brain tissues, Glioma patient cells	Down	Nrg1	(Yin et al., 2015)
Hepatocellular carcinoma (HCC)	hsa-miR-193a	HCC tumor tissues and adjacent normal tissues	Up	BMF	(Wang and Wang, 2018)
Human Embryonic Kidney cells, primary cortical neurons	hsa-miR-27a/b	HEK293, primary cortical neurons	Down	Apaf-1	(Chen et al., 2014)
Human nucleus pulposus cells	hsa-miR-155	Human nucleus pulposus cells	Down	Caspase-3	(Wang et al., 2011)
Influenza virus A infection	hsa-miR-4276	Primary human bronchial epithelial cells	Down	COX6C	(Othumpangat et al., 2014)
Laryngeal cancer	hsa-miR-34c	M4e cells	Down	BCL2	(Li et al., 2018)
Laryngeal squamous cell carcinoma	hsa-miR-221	HEp-2	Up	APAF 1	(Sun et al., 2015)
Lung cancer	hsa-miR-203	Lung cancer tissues	Up	SRC	(Wang et al., 2014)
Myocardial ischemia reperfusion	hsa-miR-15b	Adult Sprague–Dawley (SD) rats	Up	Bcl-2	(Liu et al., 2013)
Nasopharyngeal carcinoma (NPC)	hsa-miR-125b	NPC tissues	Up	A20/NF-κB signaling pathway	(Zheng et al., 2017)
Non-Hodgkin lymphoma	hsa-miR-181a	SU-DHL-4, SU-DHL-10	Up	BIM	(Lwin et al., 2010)
Non-small cell lung cancer	hsa-miR-3666	A-549		SIRT7	(Shi et al., 2016)
Non-small cell lung cancer	hsa-miR-7	Lung	Down	SMARCD1	(Hong et al., 2016)
Non-small cell lung cancer	hsa-miR-25	A549 and 95-D cells cells	Up	MOAP1	(Wu et al., 2015)
Non-small cell lung cancer	hsa-miR-34b	A549 and SPC-A-1 cells	Down	Met, p53, Mmp2	(Wang et al., 2013)
Osteoarthritis	hsa-miR-29a	Human articular cartilage samples	Up	Bax	(Miao et al., 2019)
Osteoblastoma	hsa-miR-146a	MC3T3-E1 cells	Up	Bcl2	(Zhang et al., 2017)
Osteosarcoma	hsa-miR-20	SAOS-2	Down	Fas	(Huang et al., 2012)
Pancreatic cancer	hsa-miR-365	PANC-1 and AsPC-1	Down	Bax	(Hamada et al., 2014)
Prostate cancer	hsa-miR-130a	PC-3	Down	SLAIN1	(Fujita et al., 2015)
Transitional cell carcinoma	hsa-miR-96	T24 TCC cell line and bladder TCC samples	Up	FOXO1	Guo et al., 2021

**Table 4:** CircRNAs in apoptosis.

Disease	circRNA	Tissue/Cell line	Regulation	Targeted member	References
Age-related cataract (ARC)	circHIPK3	HLECs	Down	miR-193a/CRYAA	(Liu et al., 2018)
Aortic aneursym	hsa_circ_000595	Aortic tissues/V-SMC-6110	Up	miR-19a	(Zheng et al., 2015)
Atherosclerosis	circANRIL	EC, SMC and adventitial fibroblasts (FB), HEK-293	Down	PES1	(Holdt et al., 2016)
Atherosclerosis (AS)	circRSF1	HUVECs	Down	miR-135b-5p/HDAC1	(Zhang et al., 2021)
Breast cancer	hsa_circ_0001982	MDA-MB-231, MCF-7, MDA-MB-468, MDA-MB-435s and HBL-100	Up	miR-143	(Tang et al., 2017)
Breast Cancer	hsa_circ_0001098	MCF-7	Up	miR-3942	(Zhao et al., 2018)
Cardiac senescence	circ-Foxo3	MDA-MB-231 4T1 66C14, MB-468, and MB-231		MDM2/P53	(Du et al., 2017)
Chronic obstructive pulmonary disease	hsa_circ_0006872	HPMECs and BEAS-2B	Up	miR-145-5p/NF-κB	(Xue et al., 2021)
Colorectal cancer	hsa_circ_0007534	CRC cells	Up		(Zhang et al., 2018)
Gastric cancer	circ-ZFR	GC cells	Down	miR-130a/miR-107/PTEN	(Liu et al., 2018)
Gastric cancer	hsa_circ_0067997	GC and corresponding non-cancer tissues/GSE-1, SGC-7901, MGC-803, BGC-823 and MKN28	Up	miR-515-5p/XIAP	(Zhang et al., 2019)
Intervertebral disc degeneration	circVMA21/hsa_circ_0091702	NP cells	Down	miR-200c-XIAP	(Cheng et al., 2018)
Intervertebral disc degeneration	circERCC2	Nucleus pulposus (NP) tissues/(TBHP) stimulated NP cells (NPCs)	Down	miR-182-5p	(Xie et al., 2019)
Lead-induced neurotoxicity	circRAR1	Brain tissues of mice	Up	miR-671	(Nan et al., 2017)
Lung cancer	circUBAP2	A549	Up	miR-339-5p, miR-96-3p and miR-135b-3p	(Yin et al., 2017)
Myocardial infarction (MI)	cdr1as or ciRS-7	MCM	Up	miR-7a	(Geng et al., 2016)
Non-small cell lung cancer	hsa_circ_0043256	NSCLC	Up	miR-1252/ITCH	(Tian et al., 2017)
Non-small cell lung cancer (NSCLC)	circ_0014130	H1299 and A549 and BEAS-2B	Up	miR-142-5p/IGF-1	(Wang et al., 2020)
Oral squamous cell carcinoma	circDOCK1/hsa_circ_100721	OSCC cells	Down	miR-196a-5p/BIRC3	(Wang et al., 2018)
Osteoarthritis (OA)	circRNA-9119	Cartilage samples/SW1353	Down	miR-26a	(Chen et al., 2020)
Osteosarcoma	circ-NT5C2/hsa_circ_0092509	OS cells	Up		(Liu et al., 2017)
Osteosarcoma	hsa_circ_0009910	OS cells	Up	miR-449a/IL6R	(Deng et al., 2018)
Osteosarcoma	hsa_circ_0001564	HOS and MG-63 cells	Up	miR-29c-3p	(Song and Li 2018)
Osteosarcoma	circ_0000285	hFOB 1.19, SJSA1 and U2OS	Up	miR-409-3p	(Long et al., 2020)
Prostate cancer	hsa_circ_0001445	PCa	Up		(Kong et al., 2017)
Spinal cord injury (SCI)	circ-HIPK3	AGE1.HN, PC12	Down	miR-558/DPYSL5	(Zhao et al., 2020)

**Table 5:** LncRNAs in apoptosis.

Disease	lncRNA	Tissue/Cell line	Regulation	Targeted member	References
B cell lymphoma	FAS-AS1	Granta-519 cells	Down	RBM5	(Sehgal et al., 2014)
Bladder cancer	ANRIL	T24 and EJ cells	Up		(Zhu et al., 2015)
Bladder cancer	GAS5	T24 and EJ cells	Down	EZH2	(Wang et al., 2018)
Bladder cancer	UCA1	BLZ-211 and BLS-211 cells	Up	Ets-2	(Wu et al., 2013)
Breast Cancer	GAS5	MCF7, T-47D and MDA-MB-231 cells	Up		(Pickard and Williams, 2016)
Breast cancer	OIP5-AS1	MCF-10A, SK-BR-3 and MDA-MB-231	Up	miR-216a-5p/GLO1	(Wu et al., 2021)
Cervical & colon cancer	GAS5	HeLa and HCT116 cells	Abundant	Glucocorticoid receptor	(Kino et al., 2010)
Cervical cancer	HOTAIR	SiHa, HeLa, Caski, c4-1 cells	Up	miR-143-3p	(Liu et al., 2018)
Cervical cancer	LINP1	Hela S3, SiHa and Hela cells	Up		(Wang et al., 2018)
Cholangiocarcinoma	UCA1	CCLP1 and RBE cells	Up		(Xu et al., 2017)
Colorectal adenoma	AK027294	HCT116, HCT8, and SW480 cells	Up		(Niu et al., 2016)
Colorectal cancer	DQ786243	SW620 and HT29 cells	Up		(Sun et al., 2016)
Diabetic Cardiomyopathies	H19	Diabetic rat tissue/HEK293 cells	Down	miR-675	(Li et al., 2016)
Diabetic nephropathy	PVT1	The immortalized mouse podocyte cell line mouse podocyte clone 5 (MPC5), patient samples, mice sections	Up	FOXA1	(Liu et al., 2019)
Endometrial cancer	GAS5	HHUA and JEC cells	Down	miR-103	(Guo et al., 2015)
Gastric cancer	AC113133.1	HGC-27, AGS, and SGC-7901 cells	Up	PI3K/Akt pathway	(Huang et al., 2017)
Gastric cancer	BANCR	BGC-823 and MGC-803 cells	Up	NF-κB1/miR-9.	(Zhang et al., 2015)
Gastric cancer	MEG3	HGC-27, BGC-823 and GES-1 cells	Down		(Jiao and Zhang, 2019)
Gastric cancer	MEG3	MGC-803, HGC-27, MKN-45, SGC-7901, BGC-823 and AGS cells	Down	miR-181a	(Peng et al., 2015)
Glioma	CRNDE	GSC-U87 and GSC-U251 cells	Up	miR-186	(Zheng et al., 2015)
Glioma	EGOT	U251 and U87 cells	Down		(Wu et al., 2017)
Glioma	ANCR	U87, U251, SHG44, and U118 cells	Up	EZH2	(Cheng et al., 2020)
Hepatocellular carcinoma	GOLGA2P10	MHCC-97H, QGY-7703, SK-HEP-1 and 293T cells	Up	PERK/ATF4/CHOP pathway	(Wu et al., 2020)
Hepatocellular carcinoma	PDIA3P1	HepG2, HCCLM3, Huh7, SMMC-7721, and SK-Hep1 cells	Up	p53 pathway	(Kong et al., 2017)
Ischemia/reperfusion injury	FTX	Mice cardiomyocytes	Down	miR-29b-1-5p and Bcl2l2	(Long et al., 2018)
Lung cancer	lncFOXO1	A549, H460, HCC827 and H1299 cells	Down	PI3K/AKT signaling pathway	(Ding et al., 2018)
Lung cancer	TUG1	SPC-A1 and H1299 cell lines	Up	BAX and EXH2	(Liu et al., 2017)
Lung carcinoma	Lnc_bc060912	H1299, A549 and 293T cells	Up	PARP1 and NPM1	Luo et al., 2015
Malignant melanoma	TUG1	HEMa-LP, A375, WM35, SKMEL-5, and SK-MEL-2 cell lines	Up	miR-129-5p	(Long et al., 2018)
Myocardial I/R	H19	Mouse Model of Myocardial I/RI	Down	miR-877-3p	(Li et al., 2016)
Nasopharyngeal carcinoma	LOC401317	HNE2, HNE1, and CNE2 cells	Up (After p53 overexpression)	p53	(Gong et al., 2014)
Nasopharyngeal carcinoma	ANRIL	CNE2 and HONE1 cells	Up	miR-125a	(Hu et al., 2017)
Nasopharyngeal carcinoma	HULC	SUNE and CNE-1 cells	Up		(Jiang and Liu, 2017)
Non-small cell lung cancer	lincRNA-p21	A549, H1299, H1650, and NCI-H2087 cells	Up	PUMA	(Yang et al., 2019)
Non–small cell lung cancer	TRPM2-AS	A549 and H1299 cell lines	Up	SHC1	(Ma et al., 2017)
Non–small cell lung cancer	XLOC_008466	A549 and H460 cells	Up	miR-874	(Yang et al., 2017)
Non-small cell lung cancer	PANDAR	A549, SPC-A1 and NCI-H1299 and SK-MES-1	Down	NF-YA	(Han et al., 2015)
Osteosarcoma	MEG3	MG63 cells	Down	p53	(Shi et al., 2018)
Pancreatic cancer	HOTAIR	BxPC3, MiaPaCa-2, Suit2, and PANC-1	Down (sensitive)/Up (resistant)	DR5	(Yang et al., 2017)
Pancreatic ductal adenocarcinoma	FEZF1-AS1	PANC-1, Capan-2, MIAPaCa-2, SW1990, and BxPC-3 and HPDE6-C7 cells	Up	miR-107	(Ye et al., 2018)
Parkinson’s disease	NEAT1	SH-SY5Y cells and mice models	Up	miR-124/KLF4	(Liu et al., 2020)
Polycystic ovary syndrome	lnc-CCNL1-3:1	hLGCs	Up	FOXO1	(Huang et al., 2021)
Preeclampsia	MEG3	HTR-8/SVneo, JEG-3, human amniotic epithelial cells (WISH), and human umbilical vein endothelial cells (HUVEC)	Down		(Zhang et al., 2015)
Premature ovarian insufficiency (POI)	PVT1	Granulosa cells and POI tissues	Down	Foxo3a	(Wang et al., 2021)
Prostate cancer	PCAT-1	PC3, DU145, LNCap and C4-2 cells	Up	miR-145-5p	(Xu et al., 2017)
Prostate cancer	SOCS2-AS1	LNCaP, VCaP and their LTAD cells	Up		(Misawa et al., 2016)
Rheumatoid arthritis	MALAT1	Rheumatoid arthritis fibroblastlike synoviocytes (RAFLS)	Up		(Pan et al., 2016)
T-cell acute lymphoblastic leukemia	T-ALL-RLncR1	Jurkat cells	Up	Par-4	(Zhang et al., 2014)
Urinary bladder cancer	lincRNA AATBC	UM-UC-3 and EJ bladder cancer cells	Up	JNK and NRF2 signaling pathways	(Zhao et al., 2014)
